# Homophily in grant evaluations

**DOI:** 10.1038/s41562-026-02554-w

**Published:** 2026-08-13

**Authors:** Nicola Fuchs-Schündeln, Felix Holub

**Affiliations:** 1 WZB Berlin Social Science Center, Berlin, Germany; 2 Goethe University Frankfurt, Frankfurt am Main, Germany

**Keywords:** Funding, Science, technology and society, Economics

## Abstract

Funding agencies play a central role in science by awarding grants to researchers. Grant review can be influenced by bias, including preferences for applicants who share reviewers’ gender, country or citizenship. Using 37,073 applications for European Research Council grants with multiple reviews per proposal, we show that reviewers who share an applicant’s country of work or citizenship award higher scores than reviewers with no such match. This effect is strongest when both country and citizenship align. Shared gender increases scores to a smaller extent. Further analyses indicate that these findings are unlikely to be driven by shared tastes or informational advantages. At the panel level, correlational evidence shows that applicants are more likely to advance past the first stage when the chair or more panel members share their country of work or citizenship. Country of work and citizenship may therefore be relevant sources of bias in international grant review.

## Abstract (web summary)

Fuchs-Schündeln and Holub show evidence of a homophily bias in scientific grant application review—evaluators are more favourable to applicants from their own country and, to a lesser extent, of their own gender.

## Main

Funding agencies are central to scientific progress and for academic careers. Winning competitive grants provides financial resources to conduct new research projects and influences recognition, promotions and future funding. Unlike journal peer reviewers, who assess the merits of completed research, grant evaluators must judge the future potential of projects yet to be undertaken. This uncertainty allows for greater subjectivity^1^, making the process more vulnerable to homophily, the preferential treatment of applicants who share certain characteristics with the reviewer. Especially for the most competitive and prestigious grants, even seemingly small inefficiencies in evaluation that cause some of the very best ideas to be overlooked can put the future of science on a suboptimal path.

Extensive work examines demographic disparities in grant outcomes, especially by gender and, to a lesser extent, race and ethnicity. While most studies detect no systematic gender gaps^2,3^, some exceptions exist^4^. Disparities by race and ethnicity appear more pronounced^5^. Some studies examine whether matches in reviewer and applicant gender are associated with better evaluations, with some finding no association^6,7^, while others observe that male reviewers tend to rate male applicants more favourably^8^. Shared institutional ties, kinship or supervisor relationships have also been examined, with several studies finding that such connections correlate with more favourable evaluations^9–11^.

However, this literature remains largely descriptive, documenting differential reviewer ratings or success rates. An important methodological challenge for detecting homophily lies in controlling for potential selection effects. For example, if higher-quality proposals are systematically more likely to be allocated to same-gender reviewers, observed associations could simply reflect underlying differences in proposal quality rather than same-gender homophily. This limitation largely results from the nonrandom assignment of reviewers and, in many settings, the use of joint panel decisions, which preclude within-applicant analyses. Only one study has directly addressed this challenge, namely, an early analysis of National Science Foundation grants that controls for unobserved proposal quality and finds evidence of gender-based out-group favouritism^12^. This study, however, only analyses the field of economics, uses data from the late 1980s and covers less than 1,500 proposals, with fewer than 10% coming from female applicants.

While causal evidence for homophily in grant reviews is therefore not well established, related research in other academic settings such as journal publications and academic promotions finds that shared attributes such as gender, (past) affiliation or coauthorship networks positively affect evaluation outcomes when accounting for unobserved quality^13–16^.

In this study, we examine whether homophily based on shared gender, citizenship or country of work influences grant evaluation outcomes at the European Research Council (ERC). The ERC is the leading source of competitive funding for basic research in Europe, supporting high-risk, high-return projects across all scientific disciplines. In 2024, the ERC awarded over €2.1 billion to 1,103 projects. Between 2021 and 2024, 16.2% of all research articles published in the scientific journal Nature acknowledged funding by the ERC. The ERC covers all EU member countries and some associated countries, including the UK, Switzerland and Israel. Applicants may be based anywhere, but projects must be planned to be carried out in a host institution located in an EU member or associated country.

This study makes three contributions to the literature. First, we provide causal evidence of homophily in individual ERC reviewer scores across all scientific fields by leveraging within-proposal and within-reviewer variation. Second, we introduce citizenship and country of work as new group dimensions in the literature, and find effects substantially larger than for gender. Third, we provide suggestive evidence that the observed homophily effects are unlikely to result from shared tastes or informational advantages. We complement these causal estimates with correlational evidence on panel-level decisions.

ERC calls are annual and open to researchers from any field, without restrictions on topic or methodology. The ERC offers three career stage-specific grant schemes: starting grants (2–7 years post-PhD, up to €1.5 million per grant), consolidator grants (7–12 years post-PhD, up to €2 million) and advanced grants (established researchers, up to €2.5 million).

Applications are evaluated by 1 of 27 panels grouped into 3 scientific domains: social sciences and humanities, physical sciences and engineering, and life sciences. Each panel consists of 3 groups (1 for each scheme) of approximately 15 renowned experts. Panel member identities are not disclosed until after funding rounds, except for the panel chairs.

The evaluation process consists of two steps, each involving individual evaluations followed by a panel meeting for joint decisions.

In step 1, panellists individually review a short version of each proposal, evaluating both the project and the principal investigator (PI) on a 5-point scale (1–5, in 0.5 increments, 5 best). Evaluators also provide written comments, which we do not analyse. The panel then meets and after discussions assigns each proposal a grade of A, B or C. Proposals graded A advance to the next step, while all others are rejected, with different restrictions on resubmission.

In step 2, panellists again individually review a longer version of the proposal. In addition, the panel requests reports from remote referees who are experts in the proposal’s field. These external assessments are discussed in the panel meeting and also inform the final funding decisions. Remote referees could, in principle, also exhibit homophily. We nevertheless focus on panel members for three reasons: they are the central decision-makers; the evaluation procedure for remote referees changed over the sample period; and most remote referees review only a single proposal, making it impossible to control for reviewer leniency. After conducting 30-minute panel interviews with all step 2 applicants, the panel assigns a final grade of A (recommended for funding) or B (not recommended). Since the total funds assigned to each panel are usually not enough to finance all proposals graded A, the panel also ranks these proposals, with the highest ranked proposals ultimately receiving funding. The ERC does not prescribe a specific mechanism for how panels arrive at the final ranking, and panels may therefore differ in how they aggregate opinions and reconcile disagreement. These discussions draw on the full set of materials available at that stage, including panel members’ assessments, external referee reports and the interview. As a result, ranking decisions may reflect additional considerations raised during deliberation beyond the individual numeric scores.

Our analysis covers all 37,073 ERC grant applications from 2019 to 2023. In step 1, proposals are evaluated by an average of 4.2 panel reviewers. The average project score in step 1 is 3.29 (standard deviation (s.d.) = 0.99), and the average PI score is 3.55 (s.d. = 0.90). In the panel decision, 32% of the proposals receive an A and advance. The 11,883 proposals in step 2 are evaluated by an average of 4.0 panel reviewers, complemented by 4.5 external remote referees. The average project score in step 2 is 3.90 (s.d. = 0.69), and the average PI score is 4.13 (s.d. = 0.58). At this stage, 63% of proposals receive an A, and 44% ultimately receive funding offers. For applicants and panel reviewers, we observe gender, citizenship and country of work. For country of work, the applicant’s country refers to the intended host institution, while for reviewers, it is their self-indicated country of main affiliation.

## Results

### Individual reviews

Each step of the evaluation process begins with individual reviews by panel members. We analyse reviewer-specific ratings at both stages of the process, exploiting the fact that each proposal has multiple reviewers, and each reviewer evaluates multiple proposals. We estimate the effect of demographic matches on individual ratings for both scores (project and PI) and in both steps (step 1 and step 2) using

1 $${y}_{ij}={\mu }_{i}+{\psi }_{j}+\delta \times 1({z}_{i}={z}_{j})+{\varepsilon }_{ij}.$$

Here *y*_*ij*_ is the score assigned by reviewer *j* to proposal *i*. The fixed effect (or dummy) *μ*_*i*_ captures all proposal-specific characteristics, including unobserved quality. The fixed effect *ψ*_*j*_ captures reviewer-specific tendencies, including leniency. The indicator 1(*z*_*i*_ = *z*_*j*_) equals 1 when the applicant and reviewer share a given attribute (gender, citizenship or country of work), and 0 otherwise. The coefficient *δ* measures the average difference in scores associated with such matches. Identification of *δ* comes from within-proposal and within-reviewer variation in matches, holding proposal quality and reviewer leniency constant. In essence, we compare the evaluations of the very same proposal by (i) reviewers who share an attribute with the applicant with (ii) reviewers who do not share the attribute with the applicant, all the while accounting for the general tendency of each reviewer to be more or less lenient.

#### Same-gender homophily

**Fig. 1:**
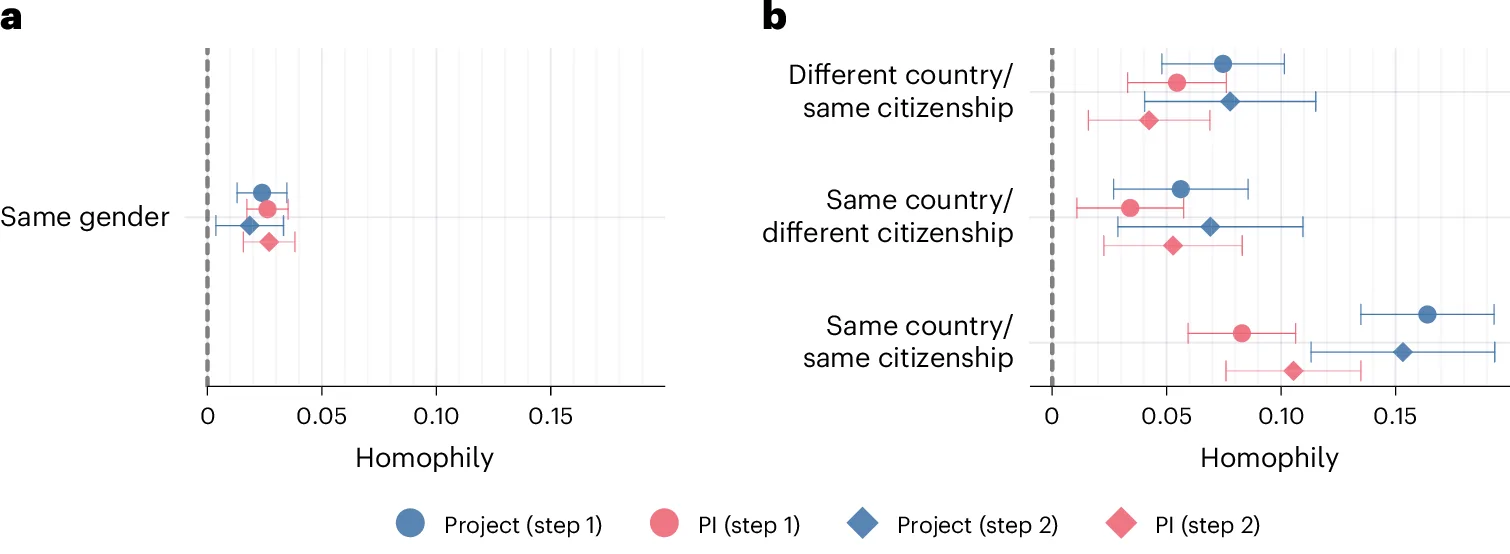
Homophily in individual reviews. **a**, Gender-based homophily. **b**, Country- and citizenship-based homophily. The unit of observation is an individual reviewer–proposal score. Each observation is one administrative review record for a proposal by a reviewer. Multiple reviews of the same proposal and multiple reviews by the same reviewer are distinct review records; inference accounts for this dependence using two-way clustering of standard errors at the project and reviewer levels. All tests of statistical significance are two-sided *t*-tests of the null hypothesis that the coefficient equals zero. No adjustment was made for multiple comparisons. The reference group in each comparison is reviewer–proposal pairs without the indicated shared characteristic. Points denote regression coefficient estimates. Error bands denote 95% CIs centred on the coefficient estimate and calculated using standard errors clustered at the project and reviewer levels. All point estimates in the same panel, colour and marker shape come from the same regression. Sample sizes, defined as the number of reviewer–proposal score observations used in each regression, are as follows: project score (step 1), *N* = 155,090 (gender), *N* = 153,736 (country/citizenship); PI score (step 1), *N* = 155,083 (gender), *N* = 153,729 (country/citizenship); project score (step 2), *N* = 47,269 (gender), *N* = 46,872 (country/citizenship); PI score (step 2), *N* = 47,267 (gender), *N* = 46,870 (country/citizenship). See Supplementary Tables 1 and 2 for fully reported results. Source data

Figure 1a indicates statistically significant but small gender homophily. Reviewers award slightly higher scores to applicants of the same gender. In step 1, project scores increase by 0.024 points (*δ*) on the 5-point scale (95% CI = (0.013, 0.035), *P* value (*P*) < 0.001) and PI scores by 0.026 points (CI = (0.017, 0.035), *P* < 0.001). In step 2, project scores (*δ* = 0.018, CI = (0.004, 0.033), *P* = 0.014) and PI scores (*δ* = 0.027, CI = (0.016, 0.038), *P* < 0.001) increase as well. While statistically significant, these estimates represent only 2.4–4.7% of the standard deviations and are thus unlikely to influence final decisions.

Supplementary Tables 3 and 4 estimate same-gender homophily separately across grant schemes and show its general prevalence. While starting grants have the smallest estimates of same-gender homophily, the effects remain statistically significant, except for step 2 project scores. By contrast, Supplementary Tables 5 and 6 reveal that same-gender homophily varies considerably across domains. The effects are most pronounced in life sciences and physical sciences and engineering, while the social sciences and humanities have very small estimated coefficients and no statistically significant effects at the 5% level.

#### Same-country and same-citizenship homophily

Figure 1b shows that both same-country and same-citizenship matches independently increase scores, with effects being on average two to three times larger than for gender. Sharing citizenship, but not country of work, increases project scores in step 1 by 0.075 points (CI = (0.048, 0.101), *P* < 0.001). The same score increases by 0.056 points (CI = (0.027, 0.086), *P* < 0.001) when sharing country, but not citizenship. The effects are even larger when both country and citizenship match simultaneously. Sharing both country and citizenship increases step 1 project scores by 0.164 points (CI = (0.135, 0.193), *P* < 0.001), representing 16% of the standard deviation. The results are qualitatively similar for the other individual scores. PI scores in step 1 increase when sharing only country (*δ* = 0.034, CI = (0.011, 0.057), *P* = 0.004), when sharing only citizenship (*δ* = 0.055, CI = (0.033, 0.076), *P* < 0.001) and when sharing both (*δ* = 0.083, CI = (0.059, 0.106), *P* < 0.001). In step 2, the magnitudes are comparable (see Supplementary Table 2 for exact numbers). Thus, homophily based on citizenship or country of work plays a quantitatively much more important role than homophily by gender.

Supplementary Tables 7–10 examine same-country and same-citizenship homophily separately by scheme and domain. Unlike gender-based homophily, same-country and same-citizenship homophily appears in all schemes and domains, with no clear pattern in the point estimates. Supplementary Table 11 shows that homophily is not driven solely by the largest countries. Aside from step 2 PI scores, where the effect is smaller, homophily in the five respective most common country/citizenship pairs is not significantly different from that in the remaining pairs.

#### Potential confounders: shared tastes and informational advantages

We next conduct two analyses to assess whether the sizable same-country and same-citizenship effects could be confounded by shared tastes or informational advantages. Both factors may be more pronounced when reviewers and applicants share country of work or citizenship and could also affect rating decisions.

If shared country or citizenship simply captures commonality of tastes, then observed homophily may reflect reviewers’ preferences for certain research topics or methodological styles more prevalent in their home research communities. To assess this explanation, we augment our baseline regressions for same-country and same-citizenship homophily with three distinct controls: academic closeness, coauthorship networks and topic match.

We measure academic closeness using SPECTER2, a large language model trained to capture semantic similarity among scientific texts^17^, calculating the cosine similarity between each proposal’s title and abstract and a given reviewer’s 20 most-cited papers^18^ (Methods). We measure coauthorship proximity using publications in the 10 years preceding the call, distinguishing direct coauthorship and shared coauthors for 1–5- and 6–10-year windows (Methods). We measure topic matches using the ERC’s keywords indicated by the applicant and the modal keyword of proposals evaluated by the reviewer (Methods).

**Fig. 2:**
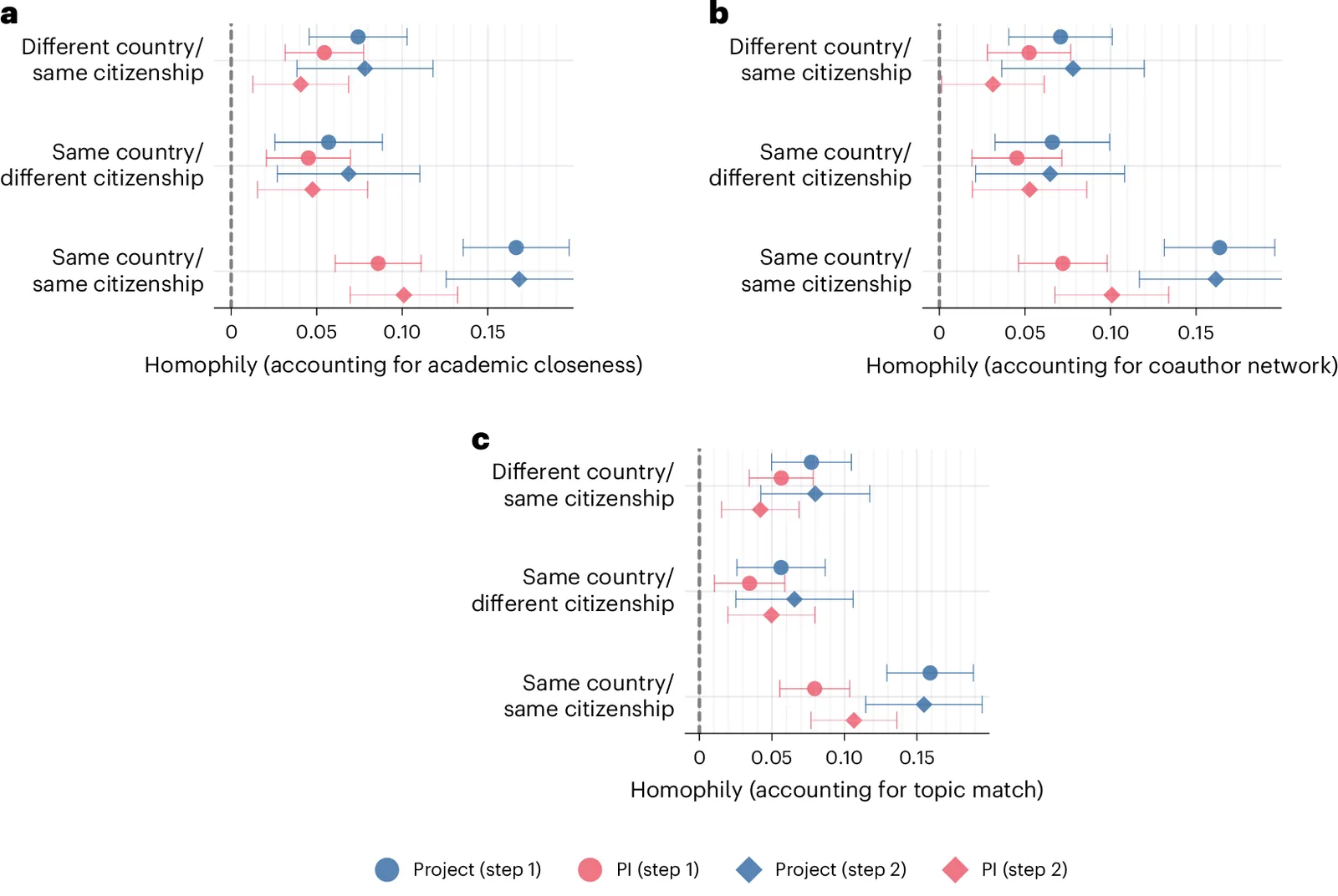
Homophily in individual reviews. **a**, Accounting for academic closeness by maximum semantic similarity. **b**, Accounting for coauthorship networks by indicators for direct coauthorship or common coauthors separately for 1–5 and 6–10 years before evaluation. **c**, Accounting for topic match by an indicator for the ERC keyword of the proposal being the reviewer’s modal keyword. The unit of observation is an individual reviewer–proposal score. Each observation is one administrative review record for a proposal by a reviewer. Multiple reviews of the same proposal and multiple reviews by the same reviewer are distinct review records; inference accounts for this dependence using two-way clustering of standard errors at the project and reviewer levels. All tests of statistical significance are two-sided *t*-tests of the null hypothesis that the coefficient equals zero. No adjustment was made for multiple comparisons. The reference group is reviewer–proposal pairs without the indicated shared country/citizenship characteristic. Points denote regression coefficient estimates. Error bands denote 95% CIs centred on the coefficient estimate and calculated using standard errors clustered at the project and reviewer levels. All point estimates in the same panel, colour and marker shape come from the same regression. Sample sizes, defined as the number of reviewer–proposal score observations used in each regression, are as follows: project score (step 1), *N* = 140,295 (closeness), *N* = 125,245 (coauthor network), *N* = 146,314 (topic match); PI score (step 1), *N* = 140,292 (closeness), *N* = 125,242 (coauthor network), *N* = 146,309 (topic match); project score (step 2), *N* = 42,747 (closeness), *N* = 38,994 (coauthor network), *N* = 46,550 (topic match); PI score (step 2), *N* = 42,746 (closeness), *N* = 38,994 (coauthor network), *N* = 46,548 (topic match). See Supplementary Tables 12–14 for fully reported results. Source data

Figure 2 shows that the coefficients on country and citizenship matches remain unchanged when including these controls, compared with the baseline results in Fig. 1b (see Supplementary Tables 12–14 for exact numbers). This makes it unlikely that the estimated country/citizenship match effects are driven by topical or intellectual proximity rather than country- and citizenship-based homophily.

If same-country or same-citizenship matches primarily capture an informational advantage, for example, because matched reviewers have more precise signals about an applicant’s track record or the feasibility of conducting the project in a given environment, then the estimated match effects need not reflect preferential treatment based on shared group membership. While we cannot directly test reviewer information sets, greater precision would be expected to be associated with more dispersion in scores, with more mass in both tails of the score distribution.

**Fig. 3:**
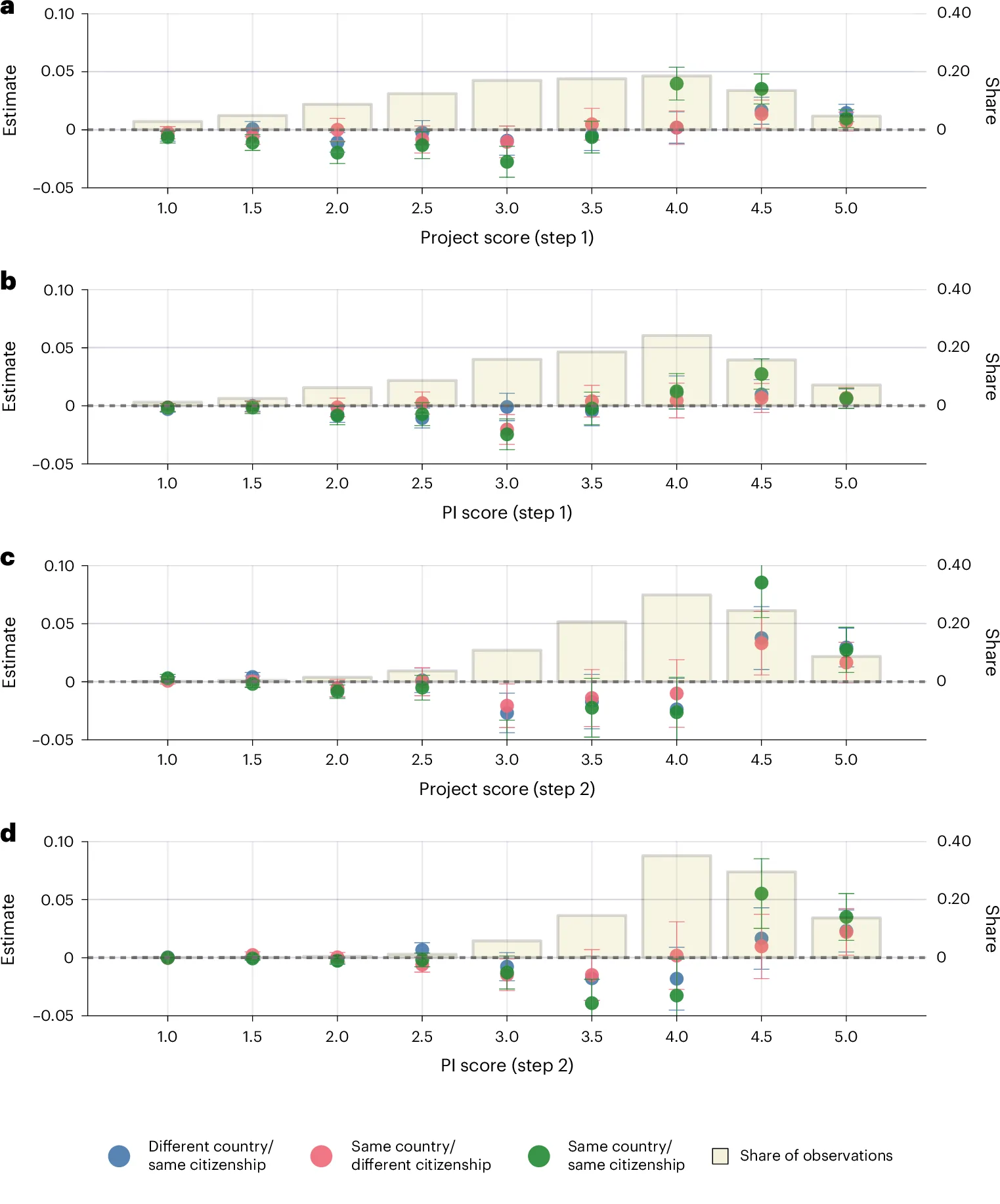
Homophily in individual reviews along the distribution. **a**, Project scores in step 1. **b**, PI scores in step 1. **c**, Project scores in step 2. **d**, PI scores in step 2. Dependent variables are indicators for whether the score is equal to the indicated value. The unit of observation is an individual reviewer–proposal score. Each observation is one administrative review record for a proposal by a reviewer. Multiple reviews of the same proposal and multiple reviews by the same reviewer are distinct review records; inference accounts for this dependence using two-way clustering of standard errors at the project and reviewer levels. All tests of statistical significance are two-sided *t*-tests of the null hypothesis that the coefficient equals zero. No adjustment was made for multiple comparisons. The reference group is reviewer–proposal pairs without shared country/citizenship. Points denote regression coefficient estimates. Error bands denote 95% CIs centred on the coefficient estimate and calculated using standard errors clustered at the project and reviewer levels. All point estimates pertaining to the same score come from the same regression. Sample sizes, defined as the number of reviewer–proposal score observations used in each regression, are as follows: project score (step 1), *N* = 153,736; PI score (step 1), *N* = 153,729; project score (step 2), *N* = 46,872; PI score (step 2), *N* = 46,870. See Supplementary Tables 15–22 for fully reported results. Source data

To test this, we re-estimate equation (1) separately for each discrete score value by replacing the dependent variable with an indicator equal to one if the score equals *s*, where *s* ∈ {1, 1.5, 2, …, 5}. Figure 3 shows that having same-country and same-citizenship reviewers systematically increases the likelihood of receiving scores at the top of the distribution, including the highest possible ratings (see Supplementary Tables 15–22 for exact numbers). For example, the probability of receiving a 5.0 as step 1 project score increases by 0.007 (CI = (−0.001, 0.015), *P* = 0.092) when only the country matches, by 0.014 (CI = (0.007, 0.022), *P* < 0.001) when only citizenship matches and by 0.010 (CI = (0.002, 0.017), *P* = 0.015) when both match, which is substantial compared with the baseline probability of 0.047. By contrast, there is little evidence that same-country and same-citizenship reviewers are more likely to assign low scores. Across both evaluation steps and criteria, positive and statistically significant effects are concentrated among the highest ratings, while coefficients near the bottom of the distribution are small and typically insignificant or even negative. For example, the probability of receiving a 1.0 as step 1 project score decreases by insignificant −0.003 (CI = (−0.008, 0.003), *P* = 0.343) when only the country of work matches, by −0.005 (CI = (−0.010, −0.001), *P* = 0.017) when only citizenship matches and by −0.006 (CI = (−0.011, −0.001), *P* = 0.013) when both match. Overall, this pattern is difficult to reconcile with precision or informational advantage being the main drivers of the homophily effects.

#### Same-rank homophily

The concern that some researchers have lower chances of securing a grant owing to fewer demographic matches with reviewers could also extend to the status of the home institution. To examine this, for each panel, we classify the ten institutions that secured the most ERC grants as elite institutions. The dummy in equation (1) equals 1 if both reviewer and applicant work at elite institutions or if both are at non-elite institutions. According to this definition, 10% of panel members and 13% of applicants work at the 10 highest-ranking elite institutions. We find no evidence of same-rank homophily. All point estimates in Supplementary Table 23 are insignificant.

### Panel decisions

Individual reviews serve as important inputs, but what matters for advancing to step 2 and securing funding are the decisions made collectively by all panel members after joint discussions. In step 1, we examine the probability of the panel assigning an A. In step 2, we examine both the probability of assigning an A and the eventual funding of the proposal.

Because each proposal receives a single panel outcome per step, we cannot include proposal fixed effects. The following panel-level estimates are therefore correlational. We relate panel outcomes (indicators for an A in step 1, and for an A and funding in step 2) and measures of demographic matches between applicants and panels:

2 $${y}_{i}={\mu }_{z(i)}+{\psi }_{p}+\delta \times f({z}_{i},{z}_{p})+{\varepsilon }_{i}$$

Here *y*_*i*_ ∈ {0, 1} is the binary panel outcome (equal to 1 if the proposal receives the respective outcome and 0 otherwise), *μ*_*z*(*i*)_ are fixed effects for the applicant’s demographic *z* (gender, country or citizenship), and *ψ*_*p*_ are panel–scheme–year fixed effects. The function *f*(*z*_*i*_, *z*_*p*_) represents either the share of panel members matching the applicant’s demographic (for example, the share of same-country panel members) or an indicator for whether the panel chair and applicant share the same demographic. Standard errors are clustered at the panel–scheme–year level.

#### Same-gender homophily in panel decisions

**Fig. 4:**
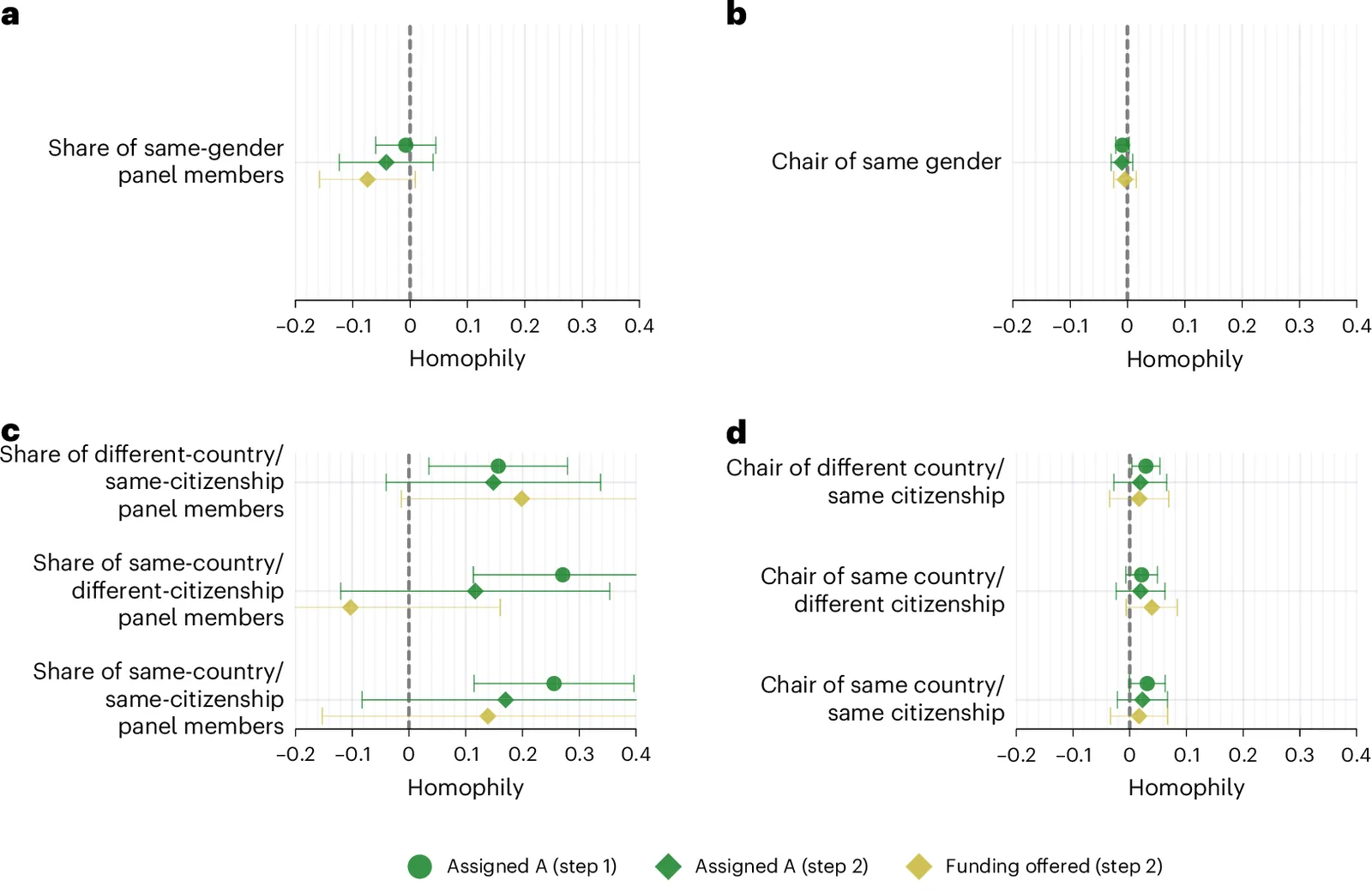
Homophily in panel decisions. **a**,**b**, Gender-based homophily by panel members (**a**) and panel chair (**b**). **c**,**d**, Country- and citizenship-based homophily by panel members (**c**) and panel chair (**d**). The unit of observation is a proposal evaluated by a panel in a scheme–year. Each observation is one administrative proposal–panel decision record. Proposals evaluated within the same panel–scheme–year may be statistically dependent; inference accounts for this dependence by clustering standard errors at the panel–scheme–year level. All tests of statistical significance are two-sided *t*-tests of the null hypothesis that the coefficient equals zero. No adjustment was made for multiple comparisons. The reference group is proposals without the indicated shared characteristic with the relevant panel composition or chair characteristic. Points denote regression coefficient estimates. Error bands denote 95% CIs centred on the coefficient estimate and calculated using standard errors clustered at the panel–scheme–year level. All point estimates in the same panel, colour and marker shape come from the same regression. Sample sizes, defined as the number of proposal–panel decision observations used in each regression, are as follows: panel shares (step 1), *N* = 37,073 (gender), *N* = 36,670 (country/citizenship); chair indicators (step 1), *N* = 37,073 (gender), *N* = 36,392 (country/citizenship); panel shares (step 2), *N* = 11,883 (gender), *N* = 11,645 (country/citizenship); chair indicators (step 2), *N* = 11,810 (gender), *N* = 11,473 (country/citizenship). See Supplementary Tables 24 and 25 for fully reported results. Source data

Figure 4a,b shows the results for the share of same-gender panel members and for the dummy indicating whether the chair shares the applicant’s gender, respectively (see Supplementary Table 24 for exact numbers). None of the estimates is statistically significant at the 5% level. However, the estimated coefficient on the share of same-gender panel members in funding decisions in step 2 is significant at the 10% level and would imply that having more reviewers of the same gender correlates with a lower funding chance (*δ* = −0.074, CI = (−0.158, 0.009), *P* = 0.081).

#### Same-country and same-citizenship homophily in panel decisions

While there is no robust evidence of homophily by panels in terms of gender—and even marginally significant evidence to the contrary—we might expect stronger correlations regarding country and citizenship, where stronger effects appear in individual reviews. Figure 4c distinguishes between the share of panel members who share (i) only citizenship with the applicant, (ii) only country of work or (iii) both. Figure 4d shows the same three matches for the panel chair (see Supplementary Table 25 for exact numbers). Figure 4c,d shows estimates separately for assigned A in step 1, assigned A in step 2 and funding offered in step 2.

In step 1, we find evidence consistent with same-country and same-citizenship homophily. For example, the coefficient on the share of same-country/same-citizenship panellists is 0.255 (CI = (0.114, 0.396), *P* < 0.001), implying that, at an average panel size of 15.1, having one additional panel member from the same country and with the same citizenship is associated with a 1.7 percentage point (p.p.) higher probability of receiving an A (baseline probability 32.6%). The corresponding chair coefficient is 0.031 (CI = (−0.001, 0.063), *P* = 0.055), implying a 3.1 p.p. higher probability of receiving an A when the chair shares both country and citizenship. The other step 1 coefficients for the shares of panel members matching only country (*δ* = 0.271, CI = (0.113, 0.428), *P* < 0.001) or only citizenship (*δ* = 0.157, CI = (0.035, 0.280), *P* = 0.012) are also positive and statistically significant. The corresponding chair coefficients are 0.021 (CI = (−0.007, 0.049), *P* = 0.137) and 0.029 (CI = (0.004, 0.053), *P* = 0.023).

In step 2, the corresponding associations are smaller and not statistically significant for either the assignment of an A or the eventual funding decision. For instance, the coefficient on the share of same-country/same-citizenship panellists is 0.170 for the assignment of an A (CI = (−0.083, 0.424), *P* = 0.187) and 0.139 for the funding outcome (CI = (−0.153, 0.431), *P* = 0.350). Across the remaining match categories, step 2 coefficients are likewise statistically insignificant; the largest point estimate is for the share of panel members who match the applicant on citizenship only in the funding outcome (*δ* = 0.199, CI = (−0.014, 0.411), *P* = 0.066). Other estimates are shown in Supplementary Table 25.

However, given the homophily-affected selection into step 2, there is an ex ante advantage for applicants with a closer match to the panel. To quantify the overall association of panel–applicant matches with funding across both steps, we combine the step 1 association with advancing to step 2 and the step 2 association with funding conditional on advancing. Concretely, we compute an average partial effect (APE) by applying the product rule to the fitted values from the step 1 and step 2 linear probability models (LPMs) (Methods and Table 1). For example, for the same-country/same-citizenship panel-member match, the APE of 15.7 p.p. results from 0.255 × 43.4% + 0.139 × 33.5%. For the average applicant to the ERC, having one additional panel member sharing both country and citizenship is associated with a 1.0 p.p. higher funding probability (CI = (0.3, 1.8), *P* = 0.007). The corresponding associations are 0.6 p.p. (CI = (−0.1, 1.4), *P* = 0.107) for 1 additional same-country/different-citizenship panellist, and 0.9 p.p. (CI = (0.3, 1.5), *P* = 0.006) for 1 additional different-country/same-citizenship panellist. Repeating the same procedure on the chair yields associations of 1.9 p.p. (CI = (−0.3, 4.2), *P* = 0.093) higher funding probability for same-country/same-citizenship, 2.4 p.p. (CI = (0.5, 4.3), *P* = 0.015) for same-country/different-citizenship and 1.9 p.p. (CI = (−0.1, 3.7), *P* = 0.070) for different-country/same-citizenship (Table 1). Given a baseline funding probability of 14.2% in our sample, these magnitudes are not negligible.

**Table 1:** APEs of panel–applicant matches and chair–applicant matches on funding probabilities

	Panel members		
	Different country/same citizenship	Same country/different citizenship	Same country/same citizenship
Step 1 coefficient ${\widehat{\delta }}^{1}$: assigned A	0.158	0.290	0.255
Step 2 coefficient ${\widehat{\delta }}^{2}$: funding offered	0.199	−0.103	0.139
Mean LPM fitted value: assigned A (in %)	33.5	33.5	33.5
Mean LPM fitted value: funding offered (in %)	43.4	43.4	43.4
APE (in p.p.)	13.5	9.1	15.7
Wild bootstrap *P*	0.006	0.107	0.007
Implied effect of +1 panel member (in p.p.)	0.9	0.6	1.0
95% CI (in p.p.)	(0.3, 1.5)	(−0.1, 1.4)	(0.3, 1.8)

	Chair		
	Different country/same citizenship	Same country/different citizenship	Same country/same citizenship
Step 1 coefficient ${\widehat{\delta }}^{1}$: assigned A	0.030	0.026	0.031
Step 2 coefficient ${\widehat{\delta }}^{2}$: funding offered	0.017	0.039	0.016
Mean LPM fitted value: assigned A (in %)	33.4	33.4	33.4
Mean LPM fitted value: funding offered (in %)	43.5	43.5	43.5
APE (in p.p.)	1.9	2.4	1.9
Wild bootstrap *P*	0.070	0.015	0.093
95% CI (in p.p.)	(−0.1, 3.7)	(0.5, 4.3)	(−0.3, 4.2)

This table shows components of the APEs (in p.p.) on the probability of receiving a funding offer. The upper panel uses match measures based on panel shares; the lower panel uses match indicators for the panel chair. Rows ‘assigned A (step 1)’ and ‘funding offered (step 2)’ show the estimated coefficients on the respective match measure from the step 1 and step 2 LPMs (Supplementary Tables 24 and 25). Rows ‘mean fitted value (assigned A)’ and ‘mean fitted value (funding offered)’ show means of the corresponding LPM fitted values. APEs are computed by combining step 1 and step 2 estimates using the product rule; see Methods for details on the construction and the evaluation sample. Statistical inference uses a wild cluster bootstrap with 999 draws, clustered at the panel–scheme–year level. Reported *P* values are two-sided wild-bootstrap tests of the null hypothesis that the corresponding APE equals zero; 95% CIs are based on the same bootstrap procedure. No adjustment was made for multiple comparisons.

To illustrate what these numbers imply, we contrast applicants from the five most represented countries by number of applications (Germany, the UK, France, Italy and Spain), which together account for just over half of all submissions, with applicants from all other countries. Table 2 shows that applicants from these top-five countries face panels in which on average 4.6% of members match both their country of work and citizenship, compared with 1.5% for applicants from all other countries. The share matching country of work but not citizenship is also higher (4.5% versus 1.8%), as is the share matching citizenship but not country (3.8% versus 2.6%). To translate these differences in panel composition into differences in predicted funding probabilities, we multiply the respective APEs by the corresponding average shares of panel members who match the applicant on (i) citizenship only, (ii) country of work only or (iii) both. This calculation implies an expected homophily-related increase in the unconditional funding probability of 1.7 p.p. for applicants from the top-five countries and 0.8 p.p. for applicants from all other countries. The implied gap between these 2 groups is 0.9 p.p., which is not negligible given that it corresponds to about 6% of the baseline success probability.

**Table 2:** Panel composition matches by applicant country of work and implied partial effects on funding

	Share of panel members who are			
	Different country/same citizenship	Same country/different citizenship	Same country/same citizenship	
Applicant working in				
Top-five country (in %)	3.8	4.5	4.6	
Other country (in %)	2.6	1.8	1.5	
Δ (top-five − other; in p.p.)	1.2	2.6	3.1	
APE (in p.p.)	13.5	9.1	15.7	
Predicted effect (APE × share)				Total
Top-five country (in p.p.)	0.5	0.4	0.7	1.7
Other country (in p.p.)	0.4	0.2	0.2	0.8
Δ (top-five − other; in p.p.)	0.2	0.2	0.5	0.9

This table shows the share of panel members who match the applicant on citizenship only, country of work only or both. Applicants are grouped by country of work: the five largest ERC application countries (Germany, UK, France, Italy and Spain) versus all others. The lower block shows APEs (in p.p.) by match type and the implied predicted effect (APE × share). Sample sizes (step 1) are *N* = 19,938 for top-five countries and *N* = 17,135 for other countries.

Supplementary Tables 26–31 show all results for panel decisions and same-country and same-citizenship homophily separately by scheme and domain. The main results are confirmed, with no clear patterns by scheme or domain.

Because panel decisions are taken collectively in the meeting and moderated by the chair, the composition of the full panel may matter beyond the assigned reviewers, particularly for borderline proposals near decision thresholds or with diverging reviews, which typically receive the most discussion time and involve the greatest discretion. Still, the opinion of the assigned reviewers likely carries the greatest weight in these discussions. As a robustness check, we therefore also estimate versions where we define match exposure using only the assigned panel reviewers. The robustness check points to the same main conclusion as before: the strongest and most robust positive association is observed when applicants and evaluators share both country of work and citizenship, whereas the country-only and citizenship-only associations are less stable (Supplementary Table 32).

We estimate equation (2) as an LPM. Doing so facilitates the inclusion of fixed effects, keeps the specification comparable to the analysis of individual reviews and allows *δ* to be directly interpreted as a change in probability (in percentage points). As a robustness check, we also estimate logit models, which yield very similar APEs and levels of statistical significance (Supplementary Table 33).

## Discussion

We show that matches in country of work and citizenship and, to a lesser extent, gender causally raise scores awarded by individual ERC panel reviewers. What ultimately matters, however, are joint panel decisions. Here we find that greater alignment between applicants and panels in terms of country and citizenship correlates with a higher chance of receiving funding.

These findings relate to a growing literature on disparities and bias in grant peer review. Previous work shows that reviewer characteristics shape evaluations. For example, reviewers with higher expertise grade more strictly^19^, and gender differences have been documented both in how applicants are evaluated and in how male and female reviewers evaluate applications^20,21^. Other recent work argues that many proposals are of very similar quality and therefore hard to compare, leading to substantial discretion^22,23^. This discretion leaves room for considerations unrelated to merit, and panel discussions may not fully correct them^24^. Within this context, we show that who reviews a proposal matters also through matches between reviewer and applicant: shared citizenship and country of work systematically increase individual reviewers’ scores even when comparing the same proposal across different reviewers and holding reviewer-specific leniency constant. Moreover, these effects are not explained by academic proximity or topical alignment, suggesting that they are unlikely to reflect differences in reviewer expertise or fit.

A main contribution of our study is methodological: we identify homophily in individual panel-member reviews using within-proposal and within-reviewer variation, which accounts for unobserved proposal quality and reviewers’ general stringency. This allows us to isolate systematic differences in how the same proposal is scored by matched versus non-matched reviewers, addressing selection concerns that limit much of the existing evidence on homophily. At the same time, our evidence on panel outcomes is correlational because each proposal receives only one panel decision per stage, which precludes proposal fixed effects. As a result, unobserved differences in proposal quality could be correlated with applicant–panel matches, for example, if some panels attract stronger proposals from particular countries and also differ in the share of panel members from those countries.

The main implication of our research is that evaluation outcomes partly depend on who reviews an application, not only on what is proposed. Because country- and citizenship-based matches are unevenly distributed across applicants, homophily can translate into systematic advantages for applicants from large or well-represented research systems and disadvantages for applicants from smaller countries. In that sense, homophily can potentially reduce efficiency and hinder the ERC’s aim of funding the best science.

While the ERC is international by design, similar dynamics may also play a role in national funding agencies. In such settings, applicants usually work in the same country. However, given the international mobility of scientists^25^, applicants and reviewers may still differ in citizenship (especially in very international research environments such as the USA or Switzerland), making citizenship-based matches potentially relevant. At the same time, reviewer–applicant competition may be more salient in national settings, so the magnitude or even direction of same-citizenship effects could differ. Other academic settings, such as publishing, are likewise highly international, with editorial boards, reviewers and authors spread across the globe.

The ERC’s multi-stage evaluation format has important implications in light of our findings. A potential advantage of staging is that later-step information can, in principle, correct idiosyncratic judgements from earlier reviews. At the same time, step 1 is a gatekeeping stage: even modest identity-related differences in individual scores can affect which proposals advance and therefore which applicants benefit from the more information-rich second stage.

Our findings suggest several policies that could potentially reduce the scope for homophily in grant review. An obvious lever is reviewer assignment. Agencies could limit same-citizenship and same-country-of-work matches, although this might not be feasible in all cases. Relatedly, increasing panel diversity could mechanically reduce the frequency of such matches and limit the influence of any single in-group perspective in deliberations. Nationality-based restrictions are used in other high-stakes settings, for example, in World Trade Organization panels, which exclude panellists from the countries concerned by the dispute. Another measure could be to require an additional assessment for proposals near the cut-off, where small score differences are most consequential. Relatedly, partial lotteries have been proposed as a way to limit the impact of somewhat arbitrary and potentially biased marginal decisions^26^. Agencies may also limit spillovers from applicant identity to project assessments by separating evaluations, for example, scoring the project on materials that omit PI identifiers where feasible and scoring the PI separately. Agencies could also consider reducing discretion by making evaluation criteria more explicit and tightly defined, which could limit opportunities for non-merit considerations.

Future research should evaluate to what extent such interventions can be effective in reducing country- or citizenship-based homophily in practice. More research is also required to quantify the cost of homophily, which implies that funds are potentially not used as efficiently as they could be, for innovation. Finally, in cooperation with funding agencies, future work could strengthen causal inference on panel-level decisions by implementing experiments, for example, by randomly assigning proposals to independent sub-panels that differ in their demographic composition.

## Methods

### SPECTER2 embeddings and academic closeness

In this study, we use SPECTER2 to measure the academic closeness between applicants and reviewers. SPECTER2 is a transformer-based large language model that creates vector representations, so-called embeddings, of scientific documents based on their text content^17^.

Compared with predecessors, SPECTER2 covers more scientific fields and is not limited to citation prediction. It is first pre-trained on more than 6 million citation triplets taken from 23 scientific fields, allowing the model to learn how scholarly documents relate through citation context. This broad training set helps SPECTER2 embeddings to generalize across many disciplines represented at the ERC, although coverage may still be better in well-represented fields. After its initial training, SPECTER2 is further improved by multi-task learning. This means that it learns to handle several tasks, including finding similar papers, classification, regression and search. To do this, SPECTER2 uses special adapters that let it generate different embeddings depending on the task. In our case, we use the proximity adapter, which is trained to capture how close two scientific papers are.

Following the set-up of SPECTER2, we use only the title and abstract for generating embeddings, rather than the full text of a paper. For each applicant, we create a SPECTER2 embedding using the proposal’s title and abstract. For reviewers, we match them to entries on the OpenAlex research platform^18^ based on name and affiliation, which we preprocess to increase the likelihood of finding a match. We successfully match 3,592 of 3,969 panel members to OpenAlex profiles. We then obtain titles and abstracts of their 20 most-cited papers since 2000. For 3,590 panel members, these entries are available on the platform.

We then calculate the cosine similarity between the applicant’s proposal and each of the reviewer’s papers. Specifically, let **a**_*i*_ be the embedding of applicant *i*’s proposal, and **b**_*jr*_ the embedding of reviewer *j*’s *r*th paper. The cosine similarity is then defined as follows:

$$\,\text{cosine similarity}\,({{\bf{a}}}_{i},{{\bf{b}}}_{jr})=\frac{{{\bf{a}}}_{i}\cdot {{\bf{b}}}_{jr}}{| {{\bf{a}}}_{i}| | {{\bf{b}}}_{jr}| }$$

This value can range from −1 to 1, with higher values meaning the documents are more semantically similar.

We aggregate the set of cosine similarities between applicant *i* and reviewer *j*, using either their mean (to capture general alignment across topics) or their maximum (to capture the closest possible connection). These variables give us a quantitative measure of academic closeness. However, the quality of these similarity measures depends on the informativeness of titles and abstracts, and some nuance may still be lost in the embedding process. We therefore interpret these measures as good proxies for the true underlying closeness.

### Coauthorship networks

As an alternative measure of academic closeness or personal connections, we account for coauthorship networks as follows.

Using data collected from the OpenAlex research data platform^18^, we obtain lists of all coauthors for both applicants and reviewers. For each individual, we create two lists of coauthors based on the year of publication: those within the 1–5 years before the call, and those within the 6–10 years before the call. Repeated coauthors may appear in both lists.

For each time span, we create two mutually exclusive indicator variables. The first is a dummy equal to one if applicant and reviewer have been coauthors in the respective period—that is, if reviewer *j* appears in *i*’s coauthor list. The second is a dummy equal to one if applicant and reviewer have not directly coauthored but have each coauthored with the same third researcher in the respective period—that is, if there exists a researcher *r* appearing in both *i*’s and *j*’s coauthor lists.

We distinguish between recent (1–5 years) and more distant (6–10 years) coauthorship relationships for several reasons. First, ERC conflict-of-interest regulations prohibit reviewers from evaluating proposals by researchers with whom they have collaborated within the past 5 years, whereas such restrictions do not apply to collaborations that occurred earlier. Nonetheless, recent coauthorship links may arise legitimately, for example, through multi-authored large-scale projects or collaborative review articles that do not entail direct personal interaction. Second, coauthorship networks from more distant periods are likely to reflect weaker academic or personal connections than recent ones.

### Topic matches

As another proxy for academic closeness and shared tastes between reviewers and applicants, we construct a measure of topic match based on ERC keywords.

When applying for an ERC grant, applicants are required to indicate one or more ERC keywords that match their proposal. These predefined keywords are panel specific and are updated each year. We use these keywords to define topics. Let *K*_*i*_ denote the set of ERC keywords chosen by applicant *i*.

To assign an ERC keyword to a reviewer, we consider all proposals they evaluate in a given year. On the basis of the idea that reviewers are most likely assigned to proposals that match their expertise, we define, for each reviewer *j* and year *t*, a modal keyword *m*_*jt*_ as the most frequent ERC keyword among the proposals evaluated by *j* in year *t*. Ties are broken at random. We do this separately by year because ERC keywords change and are sometimes relabelled across call cycles. To reduce noise, we only define *m*_*jt*_ in years in which the reviewer evaluated at least 15 proposals; otherwise, *m*_*jt*_ is set missing.

For each observed review assignment between applicant *i* and reviewer *j* in year *t*, we define a topic-match indicator *T*_*ijt*_ = 1{*m*_*jt*_ ∈ *K*_*i*_}, which equals one if any applicant keyword matches the reviewer’s modal keyword. This holds for 50.2% of all observed applicant–reviewer pairings.

### APEs of same-country and same-citizenship reviewers on the probability of funding

Panel-level regressions show that advancing to step 2 (receiving an A in step 1) is positively associated with the extent to which the panel matches the applicant on country of work and/or citizenship. In step 2, the corresponding associations with funding are weaker and often statistically insignificant. Because step 2 is reached selectively, however, a small step 2 coefficient does not imply that panel composition is unrelated to the unconditional probability of receiving funding.

To aggregate associations across both steps, we combine fitted values from the step-specific LPMs estimated in the main analysis. (Equation (2) suppresses a step index; here we use superscripts *s* ∈ {1, 2} to distinguish step 1 and step 2 outcomes, coefficients and fixed effects.) Let ${\widehat{y}}_{i}^{\,1}$ denote the fitted probability that applicant *i* receives an A in step 1, and let ${\widehat{y}}_{i}^{\,2}$ denote the fitted probability that applicant *i* is funded in step 2 conditional on reaching step 2. The implied unconditional probability of funding is

3 $${\widehat{y}}_{i}={\widehat{y}}_{i}^{\,1}\times {\widehat{y}}_{i}^{\,2}.$$

Writing the step-specific models explicitly,

$$\begin{array}{rc}{y}_{i}^{1}&={\mu }_{z(i)}^{1}+{\psi }_{p}^{1}+{\delta }^{1}f({z}_{i},{z}_{p})+{\varepsilon }_{i}^{1},\\ {y}_{i}^{2}&={\mu }_{z(i)}^{2}+{\psi }_{p}^{2}+{\delta }^{2}f({z}_{i},{z}_{p})+{\varepsilon }_{i}^{2},\end{array}$$

where *f*(*z*_*i*_, *z*_*p*_) is a match measure. Let ${\widehat{\delta }}^{1}$ and ${\widehat{\delta }}^{2}$ denote the corresponding estimated coefficients on *f*(*z*_*i*_, *z*_*p*_). By the product rule,

4 $$\frac{\partial {\widehat{y}}_{i}}{\partial f({z}_{i},{z}_{p})}={\widehat{y}}_{i}^{\,2}\,{\widehat{\delta }}^{1}+{\widehat{y}}_{i}^{\,1}\,{\widehat{\delta }}^{2}.$$

We compute this quantity for each applicant in the step 1 sample and then average across applicants to obtain the APE.

The match measures *f*(*z*_*i*_, *z*_*p*_) depend only on applicant demographics (country of work and citizenship) and panel composition. These inputs are observed for all applicants.

Constructing the step 2 fitted value ${\widehat{y}}_{i}^{\,2}$ for a step 1 applicant also requires evaluating the step 2 LPM at that applicant’s relevant fixed effect values. Because the step 2 regression includes fixed effects for applicant country–citizenship cells, ${\widehat{y}}_{i}^{\,2}$ is only defined for step 1 applicants whose country–citizenship cell is represented in the step 2 estimation sample. We therefore compute APEs on the subset of step 1 applicants with such step 2 support.

In our main specification, the match vector consists of three indicators (only country, only citizenship and both), which enter the step 1 and step 2 models jointly. We compute an APE separately for each component by taking the partial derivative with respect to that component, holding the other match indicators fixed. We report APEs for both panel-share and chair-match specifications.

For inference, we use a wild cluster bootstrap with 999 draws clustered at the panel–scheme–year level and Rademacher weights, recomputing the APE statistics in each draw.

### Software

Analyses were conducted in Julia 1.10.5 using FixedEffectModels.jl 1.11.0; text embeddings were generated in Python 3.12.4 using the SPECTER2 base model allenai/specter2_aug2023refresh_base and proximity adapter allenai/specter2_aug2023refresh, accessed through Hugging Face with transformers 4.43.4 and adapters 1.0.0.

### Reporting summary

Further information on research design is available in the Nature Portfolio Reporting Summary linked to this article.

## Supplementary information

Supplementary Information Supplementary Tables 1–33.

Reporting Summary

Peer Review File

## Source data

Source Data Figs. 1–4 Source data for all four main text figures.

## Data Availability

This research is based on data provided by the European Research Council Executive Agency (ERCEA) and is accessed exclusively within the framework of the expert contract for this assignment. In accordance with the contract’s terms and the ERCEA’s instructions, the use and disclosure of these data are subject to confidentiality obligations. As a result, the underlying data cannot be made publicly available. Any reproduction of the analyses by independent researchers would require direct authorization from the ERCEA and compliance with their data access procedures. The authors’ access to the data was strictly limited to the scope of their expert assignment. Researchers interested in accessing the data would need to contact ERCEA unit ERCEA.B.1 (head Raluca Ionescu, contact https://op.europa.eu/s/AiAG). Access is at ERCEA’s discretion and is not controlled by the authors. Source data are provided with this paper.

## Code availability

The code used for data processing, analysis and figure generation is available via figshare at https://doi.org/10.6084/m9.figshare.30789395 (ref. ^27^). The repository includes pseudo data that allow the code to be run without access to the confidential ERCEA data.
