# Supplementary material for: Homophily in grant evaluations
Source: Nat Hum Behav. 2026 Aug 13;10(10):1943–52. doi: 10.1038/s41562-026-02554-w (PMC13683047; doi:10.1038/s41562-026-02554-w)
Supplement: Supplementary file 1 [file 41562_2026_2554_MOESM1_ESM.pdf]

nature human behaviour

Supplementary information https://doi.org/10.1038/s41562-026-02554-w

Homophily in grant evaluations

In the format provided by the authors and unedited

Supplementary Information

Supplementary Table 1 Same-gender homophily in individual reviews.

	Step 1		Step 2	
	Project	PI	Project	PI
	(1)	(2)	(3)	(4)
Same gender	0.024	0.026	0.018	0.027
	(<0.001)	(<0.001)	(0.014)	(<0.001)
	[0.013, 0.035]	[0.017, 0.035]	[0.004, 0.033]	[0.016, 0.038]
Project	Yes	Yes	Yes	Yes
Reviewer	Yes	Yes	Yes	Yes
N	155,090	155,083	47,269	47,267
R^2	0.553	0.657	0.502	0.602
Within-R^2	0.000	0.000	0.000	0.001
Mean of Y	3.290	3.546	3.900	4.128
SD of Y	0.988	0.903	0.689	0.577

Dependent variables are project and PI scores, each ranging from 1 to 5. Parentheses report two-sided P-values from t-tests of the null hypothesis that the coefficient equals zero, calculated using standard errors clustered at the project and reviewer levels. The second block lists the included fixed effects.

Supplementary Table 2 Same-country and same-citizenship homophily in individual reviews.

	Step 1		Step 2	
	Project	PI	Project	PI
	(1)	(2)	(3)	(4)
Same country/				
same citz.	0.164	0.083	0.153	0.105
	(<0.001)	(<0.001)	(<0.001)	(<0.001)
	[0.135, 0.193]	[0.059, 0.106]	[0.113, 0.193]	[0.076, 0.135]
Same country/				
diff. citz.	0.056	0.034	0.069	0.053
	(<0.001)	(0.004)	(0.001)	(0.001)
	[0.027, 0.086]	[0.011, 0.057]	[0.029, 0.110]	[0.023, 0.083]
Diff. country/				
same citz.	0.075	0.055	0.078	0.042
	(<0.001)	(<0.001)	(<0.001)	(0.002)
	[0.048, 0.101]	[0.033, 0.076]	[0.040, 0.115]	[0.016, 0.069]
Project	Yes	Yes	Yes	Yes
Reviewer	Yes	Yes	Yes	Yes
Rev. country/citz.	Yes	Yes	Yes	Yes
N	153,736	153,729	46,872	46,870
R^2	0.555	0.658	0.505	0.606
Within-R^2	0.001	0.001	0.003	0.002
Mean of Y	3.289	3.546	3.899	4.128
SD of Y	0.989	0.903	0.689	0.577

Dependent variables are project and PI scores, each ranging from 1 to 5. Parentheses report two-sided P-values from t-tests of the null hypothesis that the coefficient equals zero, calculated using standard errors clustered at the project and reviewer levels. The second block lists the included fixed effects.

Supplementary Table 3 Same-gender homophily in individual reviews by grant scheme in Step 1.

	STG		COG		ADG	
	Project	PI	Project	PI	Project	PI
	(1)	(2)	(3)	(4)	(5)	(6)
Same gender	0.018	0.022	0.021	0.020	0.045	0.045
	(0.028)	(0.002)	(0.031)	(0.010)	(<0.001)	(<0.001)
	[0.002, 0.034]	[0.008, 0.036]	[0.002, 0.039]	[0.005, 0.035]	[0.020, 0.070]	[0.027, 0.063]
Project	Yes	Yes	Yes	Yes	Yes	Yes
Reviewer	Yes	Yes	Yes	Yes	Yes	Yes
N	65,521	65,518	49,110	49,110	39,914	39,910
R^2	0.552	0.643	0.550	0.654	0.574	0.697
Within-R^2	0.000	0.000	0.000	0.000	0.001	0.001
Mean of Y	3.246	3.481	3.303	3.539	3.348	3.660
SD of Y	0.994	0.913	0.978	0.896	0.988	0.885

Parentheses report two-sided P-values from t-tests of the null hypothesis that the coefficient equals zero, calculated using standard errors clustered at the project and reviewer levels. STG: Starting Grant. COG: Consolidator Grant. ADG: Advanced Grant.

Supplementary Table 4 Same-gender homophily in individual reviews by grant scheme in Step 2.

	STG		COG		ADG	
	Project	PI	Project	PI	Project	PI
	(1)	(2)	(3)	(4)	(5)	(6)
Same gender	0.008	0.015	0.024	0.043	0.037	0.029
	(0.495)	(0.089)	(0.057)	(<0.001)	(0.030)	(0.013)
	[-0.014, 0.030]	[-0.002, 0.032]	[-0.001, 0.049]	[0.024, 0.061]	[0.004, 0.070]	[0.006, 0.052]
Project	Yes	Yes	Yes	Yes	Yes	Yes
Reviewer	Yes	Yes	Yes	Yes	Yes	Yes
N	20,014	20,012	16,062	16,062	11,188	11,188
R^2	0.499	0.582	0.493	0.598	0.506	0.620
Within-R^2	0.000	0.000	0.000	0.002	0.001	0.001
Mean of Y	3.855	4.072	3.879	4.101	4.009	4.268
SD of Y	0.697	0.595	0.688	0.579	0.662	0.517

Dependent variables are project and PI scores, each ranging from 1 to 5. Parentheses report two-sided P-values from t-tests of the null hypothesis that the coefficient equals zero, calculated using standard errors clustered at the project and reviewer levels. The second block lists the included fixed effects. STG: Starting Grant. COG: Consolidator Grant. ADG: Advanced Grant.

Supplementary Table 5 Same-gender homophily in individual reviews by domain in Step 1.

	PE		LS		SH	
	Project	PI	Project	PI	Project	PI
	(1)	(2)	(3)	(4)	(5)	(6)
Same gender	0.030	0.031	0.023	0.034	0.019	0.013
	(0.002)	(<0.001)	(0.016)	(<0.001)	(0.058)	(0.108)
	[0.011, 0.049]	[0.015, 0.046]	[0.004, 0.041]	[0.019, 0.049]	[-0.001, 0.039]	[-0.003, 0.029]
Project	Yes	Yes	Yes	Yes	Yes	Yes
Reviewer	Yes	Yes	Yes	Yes	Yes	Yes
N	66,682	66,680	44,145	44,144	43,420	43,416
R^2	0.554	0.666	0.578	0.688	0.532	0.623
Within-R^2	0.000	0.000	0.000	0.001	0.000	0.000
Mean of Y	3.337	3.579	3.326	3.554	3.186	3.490
SD of Y	0.944	0.862	0.996	0.926	1.036	0.936

Dependent variables are project and PI scores, each ranging from 1 to 5. Parentheses report two-sided P-values from t-tests of the null hypothesis that the coefficient equals zero, calculated using standard errors clustered at the project and reviewer levels. The second block lists the included fixed effects. PE: Physical Sciences and Engineering. LS: Life Sciences. SH: Social Sciences and Humanities.

Supplementary Table 6 Same-gender homophily in individual reviews by domain in Step 2.

	PE		LS		SH	
	Project	PI	Project	PI	Project	PI
	(1)	(2)	(3)	(4)	(5)	(6)
Same gender	0.036	0.046	0.017	0.025	-0.001	0.007
	(0.004)	(<0.001)	(0.156)	(0.008)	(0.950)	(0.514)
	[0.012, 0.061]	[0.027, 0.065]	[-0.007, 0.041]	[0.006, 0.043]	[-0.030, 0.028]	[-0.014, 0.028]
Project	Yes	Yes	Yes	Yes	Yes	Yes
Reviewer	Yes	Yes	Yes	Yes	Yes	Yes
N	21,295	21,294	13,553	13,552	12,421	12,421
R^2	0.508	0.600	0.511	0.627	0.480	0.579
Within-R^2	0.001	0.002	0.000	0.001	0.000	0.000
Mean of Y	3.891	4.123	3.956	4.165	3.853	4.097
SD of Y	0.670	0.562	0.667	0.573	0.738	0.604

Dependent variables are project and PI scores, each ranging from 1 to 5. Parentheses report two-sided P-values from t-tests of the null hypothesis that the coefficient equals zero, calculated using standard errors clustered at the project and reviewer levels. The second block lists the included fixed effects. PE: Physical Sciences and Engineering. LS: Life Sciences. SH: Social Sciences and Humanities.

Supplementary Table 7 Same-country and same-citizenship homophily in individual reviews by grant scheme in Step 1.

	STG		COG		ADG	
	Project	PI	Project	PI	Project	PI
	(1)	(2)	(3)	(4)	(5)	(6)
Same country/ same citz.	0.136	0.079	0.174	0.079	0.184	0.085
	(<0.001)	(<0.001)	(<0.001)	(<0.001)	(<0.001)	(<0.001)
	[0.088, 0.184]	[0.039, 0.120]	[0.121, 0.228]	[0.036, 0.122]	[0.133, 0.235]	[0.047, 0.123]
Same country/ diff. citz.	0.056	0.052	0.086	0.046	0.011	-0.019
	(0.011)	(0.004)	(0.001)	(0.030)	(0.735)	(0.414)
	[0.013, 0.099]	[0.017, 0.088]	[0.034, 0.138]	[0.004, 0.087]	[-0.053, 0.075]	[-0.066, 0.027]
Diff. country/ same citz.	0.072	0.063	0.096	0.055	0.046	0.048
	(0.001)	(<0.001)	(<0.001)	(0.005)	(0.080)	(0.020)
	[0.030, 0.114]	[0.029, 0.097]	[0.049, 0.143]	[0.017, 0.094]	[-0.005, 0.098]	[0.007, 0.089]
Project	Yes	Yes	Yes	Yes	Yes	Yes
Reviewer	Yes	Yes	Yes	Yes	Yes	Yes
Rev. country/citz.	Yes	Yes	Yes	Yes	Yes	Yes
N	65,194	65,191	48,515	48,515	39,489	39,485
R^2	0.554	0.644	0.554	0.656	0.576	0.698
Within-R^2	0.001	0.001	0.002	0.001	0.002	0.001
Mean of Y	3.245	3.482	3.303	3.539	3.346	3.659
SD of Y	0.995	0.913	0.978	0.896	0.988	0.885

Dependent variables are project and PI scores, each ranging from 1 to 5. Parentheses report two-sided P-values from t-tests of the null hypothesis that the coefficient equals zero, calculated using standard errors clustered at the project and reviewer levels. The second block lists the included fixed effects. STG: Starting Grant. COG: Consolidator Grant. ADG: Advanced Grant.

Supplementary Table 8 Same-country and same-citizenship homophily in individual reviews by grant scheme in Step 2.

	STG		COG		ADG	
	Project	PI	Project	PI	Project	PI
	(1)	(2)	(3)	(4)	(5)	(6)
Same country/ same citz.	0.123	0.103	0.145	0.095	0.197	0.115
	(<0.001)	(<0.001)	(<0.001)	(<0.001)	(<0.001)	(<0.001)
	[0.055, 0.192]	[0.052, 0.155]	[0.078, 0.213]	[0.045, 0.146]	[0.123, 0.270]	[0.064, 0.166]
Same country/ diff. citz.	0.068	0.065	0.029	0.041	0.142	0.044
	(0.027)	(0.006)	(0.412)	(0.125)	(0.002)	(0.145)
	[0.008, 0.129]	[0.019, 0.111]	[-0.040, 0.099]	[-0.012, 0.094]	[0.054, 0.230]	[-0.015, 0.102]
Diff. country/ same citz.	0.030	0.038	0.092	0.036	0.147	0.062
	(0.309)	(0.091)	(0.003)	(0.093)	(<0.001)	(0.025)
	[-0.028, 0.089]	[-0.006, 0.083]	[0.030, 0.154]	[-0.006, 0.077]	[0.069, 0.225]	[0.008, 0.116]
Project	Yes	Yes	Yes	Yes	Yes	Yes
Reviewer	Yes	Yes	Yes	Yes	Yes	Yes
Rev. country/citz.	Yes	Yes	Yes	Yes	Yes	Yes
N	19,925	19,923	15,886	15,886	11,056	11,056
R^2	0.500	0.584	0.499	0.602	0.510	0.624
Within-R^2	0.001	0.002	0.002	0.001	0.007	0.004
Mean of Y	3.855	4.072	3.879	4.101	4.009	4.268
SD of Y	0.698	0.595	0.688	0.579	0.663	0.516

Dependent variables are project and PI scores, each ranging from 1 to 5. Parentheses report two-sided P-values from t-tests of the null hypothesis that the coefficient equals zero, calculated using standard errors clustered at the project and reviewer levels. The second block lists the included fixed effects. STG: Starting Grant. COG: Consolidator Grant. ADG: Advanced Grant.

Supplementary Table 9 Same-country and same-citizenship homophily in individual reviews by domain in Step 1.

	PE		LS		SH	
	Project	PI	Project	PI	Project	PI
	(1)	(2)	(3)	(4)	(5)	(6)
Same country/ same citz.	0.208	0.109	0.106	0.048	0.165	0.083
	(<0.001)	(<0.001)	(<0.001)	(0.022)	(<0.001)	(0.002)
	[0.165, 0.251]	[0.076, 0.143]	[0.055, 0.157]	[0.007, 0.089]	[0.104, 0.226]	[0.030, 0.135]
Same country/ diff. citz.	0.060	0.039	0.046	0.011	0.063	0.058
	(0.008)	(0.028)	(0.095)	(0.632)	(0.028)	(0.010)
	[0.016, 0.105]	[0.004, 0.074]	[-0.008, 0.101]	[-0.033, 0.055]	[0.007, 0.120]	[0.014, 0.102]
Diff. country/ same citz.	0.086	0.072	0.065	0.061	0.068	0.023
	(<0.001)	(<0.001)	(0.009)	(0.002)	(0.013)	(0.315)
	[0.045, 0.126]	[0.041, 0.103]	[0.016, 0.113]	[0.021, 0.100]	[0.014, 0.121]	[-0.022, 0.069]
Project	Yes	Yes	Yes	Yes	Yes	Yes
Reviewer	Yes	Yes	Yes	Yes	Yes	Yes
Rev. country/citz.	Yes	Yes	Yes	Yes	Yes	Yes
N	66,178	66,176	43,696	43,695	43,029	43,025
R^2	0.556	0.667	0.580	0.689	0.534	0.625
Within-R^2	0.002	0.001	0.001	0.000	0.001	0.001
Mean of Y	3.335	3.579	3.326	3.553	3.187	3.491
SD of Y	0.945	0.863	0.997	0.927	1.036	0.936

Parentheses report two-sided P-values from t-tests of the null hypothesis that the coefficient equals zero, calculated using standard errors clustered at the project and reviewer levels. The second block lists the included fixed effects. PE: Physical Sciences and Engineering. LS: Life Sciences. SH: Social Sciences and Humanities.

Supplementary Table 10 Same-country and same-citizenship homophily in individual reviews by domain in Step 2.

	PE		LS		SH	
	Project	PI	Project	PI	Project	PI
	(1)	(2)	(3)	(4)	(5)	(6)
Same country/ same citz.	0.195	0.105	0.111	0.134	0.127	0.069
	(<0.001)	(<0.001)	(0.002)	(<0.001)	(0.006)	(0.029)
	[0.136, 0.253]	[0.063, 0.147]	[0.042, 0.180]	[0.078, 0.189]	[0.036, 0.217]	[0.007, 0.131]
Same country/ diff. citz.	0.077	0.043	0.070	0.046	0.053	0.079
	(0.010)	(0.059)	(0.037)	(0.090)	(0.260)	(0.017)
	[0.018, 0.136]	[-0.002, 0.087]	[0.004, 0.135]	[-0.007, 0.099]	[-0.040, 0.146]	[0.014, 0.145]
Diff. country/ same citz.	0.109	0.033	0.038	0.062	0.061	0.040
	(<0.001)	(0.082)	(0.302)	(0.015)	(0.135)	(0.174)
	[0.057, 0.161]	[-0.004, 0.070]	[-0.034, 0.110]	[0.012, 0.112]	[-0.019, 0.142]	[-0.018, 0.099]
Project	Yes	Yes	Yes	Yes	Yes	Yes
Reviewer	Yes	Yes	Yes	Yes	Yes	Yes
Rev. country/citz.	Yes	Yes	Yes	Yes	Yes	Yes
N	21,141	21,140	13,427	13,426	12,304	12,304
R^2	0.513	0.604	0.513	0.629	0.483	0.583
Within-R^2	0.005	0.002	0.002	0.003	0.001	0.001
Mean of Y	3.891	4.123	3.955	4.164	3.853	4.097
SD of Y	0.671	0.562	0.667	0.574	0.738	0.603

Dependent variables are project and PI scores, each ranging from 1 to 5. Parentheses report two-sided P-values from t-tests of the null hypothesis that the coefficient equals zero, calculated using standard errors clustered at the project and reviewer levels. The second block lists the included fixed effects. PE: Physical Sciences and Engineering. LS: Life Sciences. SH: Social Sciences and Humanities.

Supplementary Table 11 Same-country and same-citizenship homophily differentiating between the five most common applicant countres and the rest.

	Step 1		Step 2	
	Project	PI	Project	PI
	(1)	(2)	(3)	(4)
Same country/ same citz.	0.133	0.095	0.133	0.160
	(<0.001)	(<0.001)	(0.001)	(<0.001)
	[0.075, 0.191]	[0.047, 0.144]	[0.053, 0.214]	[0.100, 0.220]
Same country/ diff. citz.	0.054	0.033	0.070	0.057
	(0.001)	(0.009)	(0.001)	(<0.001)
	[0.023, 0.085]	[0.008, 0.057]	[0.027, 0.112]	[0.026, 0.089]
Diff. country/ same citz.	0.079	0.055	0.079	0.047
	(<0.001)	(<0.001)	(<0.001)	(0.001)
	[0.051, 0.107]	[0.033, 0.078]	[0.040, 0.118]	[0.019, 0.075]
Top 5 × Same country/ same citz.	0.039	-0.016	0.025	-0.071
	(0.252)	(0.569)	(0.594)	(0.044)
	[-0.028, 0.106]	[-0.071, 0.039]	[-0.068, 0.119]	[-0.140, -0.002]
Top 5 × Same country/ diff. citz.	0.028	0.016	-0.004	-0.048
	(0.591)	(0.682)	(0.945)	(0.330)
	[-0.073, 0.128]	[-0.062, 0.094]	[-0.131, 0.122]	[-0.146, 0.049]
Top 5 × Diff. country/ same citz.	-0.035	-0.007	-0.007	-0.044
	(0.411)	(0.846)	(0.906)	(0.293)
	[-0.119, 0.049]	[-0.073, 0.060]	[-0.129, 0.115]	[-0.125, 0.038]
Project	Yes	Yes	Yes	Yes
Reviewer	Yes	Yes	Yes	Yes
Rev. country/citz.	Yes	Yes	Yes	Yes
N	153,736	153,729	46,872	46,870
R^2	0.555	0.658	0.505	0.606
Within-R^2	0.001	0.001	0.003	0.002
Mean of Y	3.289	3.546	3.899	4.128
SD of Y	0.989	0.903	0.689	0.577

The three interactions variables indicating Top 5 are dummies taking value 1 if the respective match variable is taking one of its five most common values. Dependent variables are project and PI scores, each ranging from 1 to 5. Parentheses report two-sided P-values from t-tests of the null hypothesis that the coefficient equals zero, calculated using standard errors clustered at the project and reviewer levels. The second block lists the included fixed effects.

Supplementary Table 12 Same-country and same-citizenship homophily in individual reviews controlling for similarity.

	Step 1		Step 2	
	Project	PI	Project	PI
	(1)	(2)	(3)	(4)
Same country/ same citz.	0.167	0.086	0.168	0.101
	(<0.001)	(<0.001)	(<0.001)	(<0.001)
	[0.136, 0.198]	[0.061, 0.111]	[0.126, 0.211]	[0.070, 0.132]
Same country/ diff. citz.	0.057	0.045	0.069	0.048
	(<0.001)	(<0.001)	(0.001)	(0.004)
	[0.026, 0.088]	[0.021, 0.070]	[0.027, 0.110]	[0.015, 0.080]
Diff. country/ same citz.	0.074	0.054	0.078	0.041
	(<0.001)	(<0.001)	(<0.001)	(0.004)
	[0.046, 0.103]	[0.032, 0.077]	[0.038, 0.118]	[0.013, 0.069]
Max similarity	-1.762	-0.128	0.836	1.521
	(<0.001)	(0.324)	(<0.001)	(<0.001)
	[-2.091, -1.432]	[-0.383, 0.127]	[0.398, 1.275]	[1.199, 1.843]
Project	Yes	Yes	Yes	Yes
Reviewer	Yes	Yes	Yes	Yes
Rev. country/citz.	Yes	Yes	Yes	Yes
N	140,295	140,292	42,747	42,746
R^2	0.569	0.669	0.524	0.621
Within-R^2	0.003	0.001	0.003	0.005
Mean of Y	3.290	3.545	3.899	4.128
SD of Y	0.988	0.904	0.689	0.578

Dependent variables are project and PI scores, each ranging from 1 to 5. Parentheses report two-sided P-values from t-tests of the null hypothesis that the coefficient equals zero, calculated using standard errors clustered at the project and reviewer levels. The second block lists the included fixed effects.

Supplementary Table 13 Same-country and same-citizenship homophily in individual reviews controlling for coauthorship networks.

	Step 1		Step 2	
	Project	PI	Project	PI
	(1)	(2)	(3)	(4)
Same country/ same citz.	0.164	0.072	0.162	0.101
	(<0.001)	(<0.001)	(<0.001)	(<0.001)
	[0.131, 0.196]	[0.046, 0.098]	[0.117, 0.206]	[0.068, 0.134]
Same country/ diff. citz.	0.066	0.045	0.065	0.053
	(<0.001)	(0.001)	(0.004)	(0.002)
	[0.032, 0.100]	[0.019, 0.072]	[0.021, 0.108]	[0.019, 0.086]
Diff. country/ same citz.	0.071	0.052	0.078	0.031
	(<0.001)	(<0.001)	(<0.001)	(0.041)
	[0.041, 0.101]	[0.028, 0.077]	[0.036, 0.120]	[0.001, 0.061]
Recent coauthors	0.021	0.077	0.041	0.098
	(0.568)	(0.005)	(0.439)	(0.015)
	[-0.051, 0.093]	[0.024, 0.130]	[-0.064, 0.147]	[0.019, 0.177]
Recent common coauthor(s)	0.010	0.023	0.068	0.055
	(0.304)	(0.001)	(<0.001)	(<0.001)
	[-0.009, 0.028]	[0.009, 0.037]	[0.043, 0.092]	[0.037, 0.072]
Past coauthors	0.072	0.084	0.161	0.150
	(0.089)	(0.004)	(0.002)	(<0.001)
	[-0.011, 0.156]	[0.026, 0.142]	[0.059, 0.263]	[0.067, 0.234]
Past common coauthor(s)	-0.004	0.010	0.037	0.029
	(0.696)	(0.212)	(0.009)	(0.004)
	[-0.025, 0.017]	[-0.006, 0.026]	[0.009, 0.064]	[0.009, 0.049]
Project	Yes	Yes	Yes	Yes
Reviewer	Yes	Yes	Yes	Yes
Rev. country/citz.	Yes	Yes	Yes	Yes
N	125,245	125,242	38,994	38,994
R^2	0.562	0.665	0.529	0.625
Within-R^2	0.002	0.001	0.005	0.005
Mean of Y	3.311	3.568	3.902	4.132
SD of Y	0.978	0.891	0.687	0.576

Dependent variables are project and PI scores, each ranging from 1 to 5. Parentheses report two-sided P-values from t-tests of the null hypothesis that the coefficient equals zero, calculated using standard errors clustered at the project and reviewer levels. The second block lists the included fixed effects. The variable 'Recent Coauthors' is equal to one if applicant and reviewer have coauthored 1–5 years prior to the year of the call. The variable Recent Common Coauthor(s) is equal to one if they have not coauthored in that period, but coauthored with the same person. The variables 'Past Coauthors' and 'Past Common Coauthor(s)' are defined equivalently for 5–10 years prior to the call.

Supplementary Table 14 Same-country and same-citizenship homophily in individual reviews controlling for topic match.

	Step 1		Step 2	
	Project	PI	Project	PI
	(1)	(2)	(3)	(4)
Same country/ same citz.	0.159	0.079	0.155	0.107
	(<0.001)	(<0.001)	(<0.001)	(<0.001)
	[0.129, 0.189]	[0.055, 0.104]	[0.115, 0.195]	[0.077, 0.136]
Same country/ diff. citz.	0.056	0.035	0.066	0.050
	(<0.001)	(0.005)	(0.001)	(0.001)
	[0.026, 0.087]	[0.010, 0.059]	[0.025, 0.106]	[0.020, 0.080]
Diff. country/ same citz.	0.077	0.056	0.080	0.042
	(<0.001)	(<0.001)	(<0.001)	(0.002)
	[0.050, 0.105]	[0.034, 0.079]	[0.042, 0.117]	[0.015, 0.069]
Same topic	-0.008	-0.002	0.032	0.027
	(0.200)	(0.749)	(<0.001)	(<0.001)
	[-0.020, 0.004]	[-0.011, 0.008]	[0.016, 0.048]	[0.015, 0.039]
Project	Yes	Yes	Yes	Yes
Reviewer	Yes	Yes	Yes	Yes
Rev. country/citz.	Yes	Yes	Yes	Yes
N	146,314	146,309	46,550	46,548
R^2	0.564	0.667	0.507	0.607
Within-R^2	0.001	0.001	0.003	0.003
Mean of Y	3.295	3.549	3.899	4.127
SD of Y	0.989	0.904	0.688	0.577

Dependent variables are project and PI scores, each ranging from 1 to 5. Parentheses report two-sided P-values from t-tests of the null hypothesis that the coefficient equals zero, calculated using standard errors clustered at the project and reviewer levels. The second block lists the included fixed effects. The variable 'Same Topic' is a dummy variable equal to one if the applicant's chosen ERC keyword matches the keyword most frequently appearing in the set of proposals assigned to that reviewer in the same call year. This variable is defined only for reviewers who conducted at least 15 reviews in that year.

Supplementary Table 15 Same-country and same-citizenship homophily in project scores along the distribution in Step 1 (Scores 1.0-3.0).

	Project Score				
	= 1.0	= 1.5	= 2.0	= 2.5	= 3.0
	(1)	(2)	(3)	(4)	(5)
Diff. country/ same citz.	-0.005	0.001	-0.011	-0.002	-0.009
	(0.017)	(0.838)	(0.012)	(0.643)	(0.143)
	[-0.010, -0.001]	[-0.006, 0.007]	[-0.019, -0.002]	[-0.013, 0.008]	[-0.022, 0.003]
Same country/ diff. citz.	-0.003	-0.006	-0.000	-0.008	-0.010
	(0.343)	(0.112)	(0.995)	(0.159)	(0.132)
	[-0.008, 0.003]	[-0.013, 0.001]	[-0.010, 0.010]	[-0.020, 0.003]	[-0.024, 0.003]
Same country/ same citz.	-0.006	-0.011	-0.020	-0.013	-0.028
	(0.013)	(0.001)	(<0.001)	(0.024)	(<0.001)
	[-0.011, -0.001]	[-0.018, -0.004]	[-0.029, -0.010]	[-0.025, -0.002]	[-0.041, -0.014]
Project	Yes	Yes	Yes	Yes	Yes
Reviewer	Yes	Yes	Yes	Yes	Yes
Rev. country/citz.	Yes	Yes	Yes	Yes	Yes
N	153,736	153,736	153,736	153,736	153,736
R^2	0.417	0.348	0.326	0.312	0.295
Within-R^2	0.000	0.000	0.000	0.000	0.000
Mean of Y	0.028	0.049	0.087	0.124	0.170
SD of Y	0.166	0.216	0.282	0.330	0.375

Dependent variables are indicators for whether the score is equal to indicated value. Parentheses report two-sided P-values from t-tests of the null hypothesis that the coefficient equals zero, calculated using standard errors clustered at the project and reviewer levels. The second block lists the included fixed effects.

Supplementary Table 16 Same-country and same-citizenship homophily in project scores along the distribution in Step 1 (Scores 3.0-5.0).

	Project Score			
	= 3.5	= 4.0	= 4.5	= 5.0
	(1)	(2)	(3)	(4)
Diff. country/ same citz.	-0.005	0.002	0.016	0.014
	(0.426)	(0.789)	(0.006)	(<0.001)
	[-0.018, 0.008]	[-0.012, 0.015]	[0.005, 0.028]	[0.007, 0.022]
Same country/ diff. citz.	0.005	0.002	0.014	0.007
	(0.499)	(0.801)	(0.029)	(0.092)
	[-0.009, 0.018]	[-0.013, 0.016]	[0.001, 0.026]	[-0.001, 0.015]
Same country/ same citz.	-0.006	0.040	0.035	0.010
	(0.364)	(<0.001)	(<0.001)	(0.015)
	[-0.020, 0.007]	[0.026, 0.054]	[0.022, 0.048]	[0.002, 0.017]
Project	Yes	Yes	Yes	Yes
Reviewer	Yes	Yes	Yes	Yes
Rev. country/citz.	Yes	Yes	Yes	Yes
N	153,736	153,736	153,736	153,736
R^2	0.296	0.317	0.339	0.346
Within-R^2	0.000	0.000	0.000	0.000
Mean of Y	0.175	0.185	0.135	0.047
SD of Y	0.380	0.388	0.342	0.211

Dependent variables are indicators for whether the score is equal to indicated value. Parentheses report two-sided P-values from t-tests of the null hypothesis that the coefficient equals zero, calculated using standard errors clustered at the project and reviewer levels. The second block lists the included fixed effects.

Supplementary Table 17 Same-country and same-citizenship homophily in PI scores along the distribution in Step 1 (Scores 1.0-3.0).

	PI Score				
	= 1.0	= 1.5	= 2.0	= 2.5	= 3.0
	(1)	(2)	(3)	(4)	(5)
Diff. country/ same citz.	-0.003	-0.000	-0.008	-0.011	-0.001
	(0.034)	(0.924)	(0.030)	(0.015)	(0.856)
	[-0.006, -0.000]	[-0.005, 0.004]	[-0.014, -0.001]	[-0.019, -0.002]	[-0.013, 0.011]
Same country/ diff. citz.	-0.001	-0.001	-0.001	0.002	-0.020
	(0.394)	(0.770)	(0.729)	(0.645)	(0.002)
	[-0.005, 0.002]	[-0.006, 0.004]	[-0.009, 0.007]	[-0.007, 0.012]	[-0.033, -0.008]
Same country/ same citz.	-0.002	-0.002	-0.009	-0.007	-0.025
	(0.311)	(0.534)	(0.032)	(0.146)	(<0.001)
	[-0.005, 0.002]	[-0.007, 0.003]	[-0.016, -0.001]	[-0.017, 0.003]	[-0.038, -0.011]
Project	Yes	Yes	Yes	Yes	Yes
Reviewer	Yes	Yes	Yes	Yes	Yes
Rev. country/citz.	Yes	Yes	Yes	Yes	Yes
N	153,729	153,729	153,729	153,729	153,729
R^2	0.397	0.357	0.365	0.337	0.324
Within-R^2	0.000	0.000	0.000	0.000	0.000
Mean of Y	0.012	0.025	0.062	0.087	0.159
SD of Y	0.109	0.155	0.241	0.281	0.366

Dependent variables are indicators for whether the score is equal to indicated value. Parentheses report two-sided P-values from t-tests of the null hypothesis that the coefficient equals zero, calculated using standard errors clustered at the project and reviewer levels. The second block lists the included fixed effects.

Supplementary Table 18 Same-country and same-citizenship homophily in PI scores along the distribution in Step 1 (Scores 3.5-5.0).

	PI Score			
	= 3.5	= 4.0	= 4.5	= 5.0
	(1)	(2)	(3)	(4)
Diff. country/ same citz.	-0.005	0.011	0.010	0.006
	(0.480)	(0.117)	(0.134)	(0.169)
	[-0.017, 0.008]	[-0.003, 0.026]	[-0.003, 0.022]	[-0.003, 0.014]
Same country/ diff. citz.	0.004	0.005	0.007	0.007
	(0.577)	(0.554)	(0.298)	(0.141)
	[-0.010, 0.017]	[-0.010, 0.019]	[-0.006, 0.019]	[-0.002, 0.016]
Same country/ same citz.	-0.002	0.012	0.027	0.006
	(0.748)	(0.109)	(<0.001)	(0.151)
	[-0.016, 0.012]	[-0.003, 0.028]	[0.014, 0.040]	[-0.002, 0.015]
Project	Yes	Yes	Yes	Yes
Reviewer	Yes	Yes	Yes	Yes
Rev. country/citz.	Yes	Yes	Yes	Yes
N	153,729	153,729	153,729	153,729
R^2	0.310	0.334	0.370	0.436
Within-R^2	0.000	0.000	0.000	0.000
Mean of Y	0.185	0.241	0.157	0.071
SD of Y	0.389	0.428	0.364	0.257

Dependent variables are indicators for whether the score is equal to indicated value. Parentheses report two-sided P-values from t-tests of the null hypothesis that the coefficient equals zero, calculated using standard errors clustered at the project and reviewer levels. The second block lists the included fixed effects.

Supplementary Table 19 Same-country and same-citizenship homophily in project scores along the distribution in Step 2 (Scores 1.0-3.0).

	Project Score				
	= 1.0	= 1.5	= 2.0	= 2.5	= 3.0
	(1)	(2)	(3)	(4)	(5)
Diff. country/ same citz.	0.003	0.004	-0.007	0.001	-0.027
	(0.009)	(0.041)	(0.040)	(0.822)	(0.002)
	[0.001, 0.004]	[0.000, 0.008]	[-0.013, -0.000]	[-0.009, 0.012]	[-0.044, -0.010]
Same country/ diff. citz.	0.001	0.000	-0.005	-0.000	-0.021
	(0.600)	(0.927)	(0.152)	(0.994)	(0.031)
	[-0.002, 0.003]	[-0.004, 0.005]	[-0.013, 0.002]	[-0.012, 0.012]	[-0.039, -0.002]
Same country/ same citz.	0.003	-0.002	-0.009	-0.005	-0.051
	(0.062)	(0.227)	(0.004)	(0.343)	(<0.001)
	[-0.000, 0.006]	[-0.005, 0.001]	[-0.014, -0.003]	[-0.016, 0.005]	[-0.070, -0.033]
Project	Yes	Yes	Yes	Yes	Yes
Reviewer	Yes	Yes	Yes	Yes	Yes
Rev. country/citz.	Yes	Yes	Yes	Yes	Yes
N	46,872	46,872	46,872	46,872	46,872
R^2	0.366	0.365	0.369	0.372	0.382
Within-R^2	0.000	0.000	0.000	0.000	0.001
Mean of Y	0.001	0.004	0.015	0.037	0.108
SD of Y	0.033	0.065	0.121	0.188	0.310

Dependent variables are indicators for whether the score is equal to indicated value. Parentheses report two-sided P-values from t-tests of the null hypothesis that the coefficient equals zero, calculated using standard errors clustered at the project and reviewer levels. The second block lists the included fixed effects.

Supplementary Table 20 Same-country and same-citizenship homophily in project scores along the distribution in Step 2 (Scores 3.5-5.0).

	Project Score			
	= 3.5	= 4.0	= 4.5	= 5.0
	(1)	(2)	(3)	(4)
Diff. country/ same citz.	-0.017	-0.024	0.037	0.029
	(0.150)	(0.080)	(0.007)	(0.001)
	[-0.040, 0.006]	[-0.050, 0.003]	[0.010, 0.064]	[0.013, 0.046]
Same country/ diff. citz.	-0.014	-0.010	0.033	0.016
	(0.259)	(0.493)	(0.018)	(0.063)
	[-0.039, 0.010]	[-0.039, 0.019]	[0.006, 0.060]	[-0.001, 0.034]
Same country/ same citz.	-0.022	-0.026	0.085	0.027
	(0.081)	(0.087)	(<0.001)	(0.006)
	[-0.048, 0.003]	[-0.056, 0.004]	[0.055, 0.115]	[0.008, 0.047]
Project	Yes	Yes	Yes	Yes
Reviewer	Yes	Yes	Yes	Yes
Rev. country/citz.	Yes	Yes	Yes	Yes
N	46,872	46,872	46,872	46,872
R^2	0.376	0.364	0.388	0.438
Within-R^2	0.000	0.000	0.001	0.001
Mean of Y	0.206	0.298	0.244	0.087
SD of Y	0.404	0.458	0.430	0.281

Dependent variables are indicators for whether the score is equal to indicated value. Parentheses report two-sided P-values from t-tests of the null hypothesis that the coefficient equals zero, calculated using standard errors clustered at the project and reviewer levels. The second block lists the included fixed effects.

Supplementary Table 21 Same-country and same-citizenship homophily in PI scores along the distribution in Step 2 (Scores 1.0-3.0).

	PI Score				
	= 1.0	= 1.5	= 2.0	= 2.5	= 3.0
	(1)	(2)	(3)	(4)	(5)
Diff. country/ same citz.	0.000	-0.000	-0.003	0.007	-0.008
	(0.201)	(0.981)	(0.123)	(0.037)	(0.208)
	[-0.000, 0.001]	[-0.002, 0.001]	[-0.006, 0.001]	[0.000, 0.013]	[-0.020, 0.004]
Same country/ diff. citz.	-0.000	0.002	0.000	-0.006	-0.015
	(0.350)	(0.110)	(0.901)	(0.063)	(0.034)
	[-0.001, 0.000]	[-0.001, 0.005]	[-0.004, 0.004]	[-0.012, 0.000]	[-0.028, -0.001]
Same country/ same citz.	-0.000	-0.001	-0.003	-0.002	-0.013
	(0.502)	(0.362)	(0.047)	(0.513)	(0.078)
	[-0.001, 0.000]	[-0.002, 0.001]	[-0.005, -0.000]	[-0.008, 0.004]	[-0.027, 0.001]
Project	Yes	Yes	Yes	Yes	Yes
Reviewer	Yes	Yes	Yes	Yes	Yes
Rev. country/citz.	Yes	Yes	Yes	Yes	Yes
N	46,870	46,870	46,870	46,870	46,870
R^2	0.438	0.366	0.364	0.376	0.409
Within-R^2	0.000	0.000	0.000	0.000	0.000
Mean of Y	0.000	0.001	0.004	0.011	0.057
SD of Y	0.012	0.024	0.062	0.106	0.233

Dependent variables are indicators for whether the score is equal to indicated value. Parentheses report two-sided P-values from t-tests of the null hypothesis that the coefficient equals zero, calculated using standard errors clustered at the project and reviewer levels. The second block lists the included fixed effects.

Supplementary Table 22 Same-country and same-citizenship homophily in PI scores along the distribution in Step 2 (Scores 3.5-5.0).

	PI Score			
	= 3.5	= 4.0	= 4.5	= 5.0
	(1)	(2)	(3)	(4)
Diff. country/ same citz.	-0.018	-0.018	0.016	0.023
	(0.067)	(0.188)	(0.222)	(0.013)
	[-0.037, 0.001]	[-0.045, 0.009]	[-0.010, 0.043]	[0.005, 0.041]
Same country/ diff. citz.	-0.015	0.002	0.010	0.022
	(0.180)	(0.907)	(0.494)	(0.031)
	[-0.037, 0.007]	[-0.027, 0.031]	[-0.018, 0.037]	[0.002, 0.042]
Same country/ same citz.	-0.039	-0.033	0.055	0.035
	(<0.001)	(0.041)	(<0.001)	(0.001)
	[-0.060, -0.019]	[-0.064, -0.001]	[0.025, 0.085]	[0.015, 0.055]
Project	Yes	Yes	Yes	Yes
Reviewer	Yes	Yes	Yes	Yes
Rev. country/citz.	Yes	Yes	Yes	Yes
N	46,870	46,870	46,870	46,870
R^2	0.413	0.398	0.415	0.524
Within-R^2	0.000	0.000	0.000	0.001
Mean of Y	0.145	0.351	0.295	0.137
SD of Y	0.352	0.477	0.456	0.343

Dependent variables are indicators for whether the score is equal to indicated value. Parentheses report two-sided P-values from t-tests of the null hypothesis that the coefficient equals zero, calculated using standard errors clustered at the project and reviewer levels. The second block lists the included fixed effects.

Supplementary Table 23 Same-rank homophily in individual reviews.

	Step 1		Step 2	
	Project	PI	Project	PI
	(1)	(2)	(3)	(4)
Same institution rank (top 10)	0.014	0.013	-0.005	-0.010
	(0.227)	(0.150)	(0.705)	(0.290)
	[-0.009, 0.037]	[-0.005, 0.031]	[-0.031, 0.021]	[-0.029, 0.009]
Project	Yes	Yes	Yes	Yes
Reviewer	Yes	Yes	Yes	Yes
Rev. country	Yes	Yes	Yes	Yes
N	153,578	153,571	46,825	46,823
R^2	0.554	0.658	0.504	0.605
Within-R^2	0.000	0.000	0.000	0.000
Mean of Y	3.289	3.546	3.899	4.128
SD of Y	0.989	0.903	0.689	0.577

Dependent variables are project and PI scores, each ranging from 1 to 5. Parentheses report two-sided P-values from t-tests of the null hypothesis that the coefficient equals zero, calculated using standard errors clustered at the project and reviewer levels. The second block lists the included fixed effects.

Supplementary Table 24 Same-gender homophily in panel decisions.

	Step 1		Step 2			
	Assigned A				Funding Offered	
	(1)	(2)	(3)	(4)	(5)	(6)
Share of same gender panelists	-0.007		-0.041		-0.074	
	(0.781)		(0.320)		(0.082)	
	[-0.060, 0.045]		[-0.123, 0.040]		[-0.158, 0.009]	
Same gender chair		-0.009		-0.009		-0.004
		(0.155)		(0.328)		(0.679)
		[-0.020, 0.003]		[-0.028, 0.009]		[-0.024, 0.016]
App. gender	Yes	Yes	Yes	Yes	Yes	Yes
Panel-Scheme-Year	Yes	Yes	Yes	Yes	Yes	Yes
N	37,073	37,073	11,883	11,810	11,883	11,810
R^2	0.017	0.017	0.109	0.109	0.017	0.017
Within-R^2	0.000	0.000	0.000	0.000	0.000	0.000
Mean of Y	0.324	0.324	0.633	0.633	0.444	0.444
SD of Y	0.468	0.468	0.482	0.482	0.497	0.497

Dependent variables are indicators for receiving an A and for funding offered. Parentheses report two-sided P-values from t-tests of the null hypothesis that the coefficient equals zero, calculated using standard errors clustered at the panel-scheme-year level. The second block lists the included fixed effects.

Supplementary Table 25 Same-country and same-citizenship homophily in panel decisions.

	Step 1	Step 2				
	Assigned A				Funding Offered	
	(1)	(2)	(3)	(4)	(5)	(6)
Share of same country/ same citz. panelists	0.255		0.170		0.139	
	(<0.001)		(0.188)		(0.350)	
	[0.114, 0.396]		[-0.083, 0.424]		[-0.153, 0.431]	
Share of same country/ diff. citz. panelists	0.271		0.117		-0.103	
	(0.001)		(0.334)		(0.446)	
	[0.113, 0.428]		[-0.120, 0.354]		[-0.367, 0.162]	
Share of diff. country/ same citz. panelists	0.157		0.149		0.199	
	(0.012)		(0.123)		(0.067)	
	[0.035, 0.280]		[-0.040, 0.338]		[-0.014, 0.411]	
Same country/ same citz. chair		0.031		0.022		0.016
		(0.056)		(0.319)		(0.520)
		[-0.001, 0.063]		[-0.022, 0.067]		[-0.034, 0.067]
Same country/ diff. citz. chair		0.021		0.019		0.039
		(0.138)		(0.386)		(0.092)
		[-0.007, 0.049]		[-0.024, 0.062]		[-0.006, 0.084]
Diff. country/ same citz. chair		0.029		0.019		0.017
		(0.023)		(0.431)		(0.530)
		[0.004, 0.053]		[-0.028, 0.065]		[-0.035, 0.069]
Panel-Scheme-Year	Yes	Yes	Yes	Yes	Yes	Yes
App. country/citz.	Yes	Yes	Yes	Yes	Yes	Yes
N	36,670	36,392	11,645	11,473	11,645	11,473
R^2	0.078	0.078	0.159	0.160	0.070	0.071
Within-R^2	0.001	0.000	0.000	0.000	0.001	0.000
Mean of Y	0.326	0.326	0.634	0.634	0.445	0.445
SD of Y	0.469	0.469	0.482	0.482	0.497	0.497

Dependent variables are indicators for receiving an A and for funding offered. Parentheses report two-sided P-values from t-tests of the null hypothesis that the coefficient equals zero, calculated using standard errors clustered at the panel-scheme-year level. The second block lists the included fixed effects.

Supplementary Table 26 Same-country and same-citizenship homophily in panel decisions by grant scheme in Step 1.

	STG		COG		ADG	
	Assigned A					
	(1)	(2)	(3)	(4)	(5)	(6)
Share of same country/ same citz. panelists	0.221		0.403		0.139	
	(0.086)		(0.002)		(0.240)	
	[-0.032, 0.474]		[0.145, 0.660]		[-0.094, 0.373]	
Share of same country/ diff. citz. panelists	0.257		0.461		0.164	
	(0.044)		(0.002)		(0.336)	
	[0.007, 0.507]		[0.175, 0.747]		[-0.172, 0.499]	
Share of diff. country/ same citz. panelists	0.156		0.242		0.097	
	(0.114)		(0.027)		(0.443)	
	[-0.038, 0.350]		[0.028, 0.456]		[-0.153, 0.348]	
Same country/ same citz. chair		0.035		0.059		-0.010
		(0.180)		(0.091)		(0.647)
		[-0.016, 0.086]		[-0.009, 0.127]		[-0.055, 0.035]
Same country/ diff. citz. chair		0.016		0.023		0.038
		(0.466)		(0.336)		(0.245)
		[-0.027, 0.060]		[-0.024, 0.070]		[-0.026, 0.102]
Diff. country/ same citz. chair		0.034		0.046		-0.005
		(0.054)		(0.049)		(0.847)
		[-0.001, 0.068]		[0.000, 0.092]		[-0.056, 0.046]
Panel-Scheme-Year	Yes	Yes	Yes	Yes	Yes	Yes
App. country/citz.	Yes	Yes	Yes	Yes	Yes	Yes
N	15,490	15,490	11,443	11,231	9,271	9,204
R^2	0.094	0.094	0.097	0.096	0.096	0.097
Within-R^2	0.001	0.000	0.002	0.001	0.000	0.000
Mean of Y	0.328	0.328	0.352	0.351	0.298	0.298
SD of Y	0.469	0.469	0.477	0.477	0.457	0.457

Dependent variable is an indicator for receiving an A. Parentheses report two-sided P-values from t-tests of the null hypothesis that the coefficient equals zero, calculated using standard errors clustered at the panel-scheme-year level. The second block lists the included fixed effects. STG: Starting Grant. COG: Consolidator Grant. ADG: Advanced Grant.

Supplementary Table 27 Same-country and same-citizenship homophily in panel decisions (assigned A) by grant scheme in Step 2.

	STG		COG		ADG	
	Assigned A					
	(1)	(2)	(3)	(4)	(5)	(6)
Share of same country/ same citz. panelists	0.135		0.293		0.070	
	(0.557)		(0.170)		(0.783)	
	[-0.317, 0.586]		[-0.127, 0.713]		[-0.434, 0.575]	
Share of same country/ diff. citz. panelists	0.192		0.015		0.091	
	(0.276)		(0.951)		(0.718)	
	[-0.155, 0.539]		[-0.471, 0.501]		[-0.405, 0.586]	
Share of diff. country/ same citz. panelists	0.265		0.103		0.091	
	(0.111)		(0.486)		(0.661)	
	[-0.062, 0.592]		[-0.189, 0.395]		[-0.318, 0.499]	
Same country/ same citz. chair		0.021		-0.017		0.077
		(0.573)		(0.655)		(0.058)
		[-0.052, 0.093]		[-0.093, 0.059]		[-0.003, 0.157]
Same country/ diff. citz. chair		-0.007		0.021		0.044
		(0.837)		(0.623)		(0.248)
		[-0.077, 0.062]		[-0.062, 0.104]		[-0.031, 0.120]
Diff. country/ same citz. chair		0.012		-0.007		0.118
		(0.777)		(0.826)		(0.022)
		[-0.072, 0.097]		[-0.071, 0.057]		[0.017, 0.219]
Panel-Scheme-Year	Yes	Yes	Yes	Yes	Yes	Yes
App. country/citz.	Yes	Yes	Yes	Yes	Yes	Yes
N	4,842	4,797	3,865	3,787	2,663	2,612
R^2	0.156	0.155	0.199	0.199	0.186	0.189
Within-R^2	0.001	0.000	0.001	0.000	0.000	0.003
Mean of Y	0.609	0.610	0.645	0.642	0.667	0.668
SD of Y	0.488	0.488	0.479	0.480	0.471	0.471

Dependent variables are indicators for receiving an A. Parentheses report two-sided P-values from t-tests of the null hypothesis that the coefficient equals zero, calculated using standard errors clustered at the panel-scheme-year level. The second block lists the included fixed effects. STG: Starting Grant. COG: Consolidator Grant. ADG: Advanced Grant.

Supplementary Table 28 Same-country and same-citizenship homophily in panel decisions (funding offered) by grant scheme in Step 2.

	STG		COG		ADG	
	Funding Offered					
	(1)	(2)	(3)	(4)	(5)	(6)
Share of same country/ same citz. panelists	0.073		0.199		0.140	
	(0.770)		(0.439)		(0.620)	
	[-0.420, 0.565]		[-0.309, 0.707]		[-0.417, 0.697]	
Share of same country/ diff. citz. panelists	-0.004		-0.272		-0.096	
	(0.984)		(0.304)		(0.766)	
	[-0.396, 0.388]		[-0.792, 0.249]		[-0.731, 0.540]	
Share of diff. country/ same citz. panelists	0.182		0.214		0.236	
	(0.280)		(0.250)		(0.356)	
	[-0.150, 0.515]		[-0.153, 0.582]		[-0.268, 0.740]	
Same country/ same citz. chair		-0.014		0.014		0.064
		(0.741)		(0.706)		(0.250)
		[-0.099, 0.071]		[-0.058, 0.086]		[-0.046, 0.174]
Same country/ diff. citz. chair		0.001		0.055		0.076
		(0.975)		(0.205)		(0.162)
		[-0.071, 0.073]		[-0.031, 0.141]		[-0.031, 0.183]
Diff. country/ same citz. chair		0.035		-0.042		0.080
		(0.420)		(0.329)		(0.124)
		[-0.051, 0.121]		[-0.126, 0.043]		[-0.022, 0.183]
Panel-Scheme-Year	Yes	Yes	Yes	Yes	Yes	Yes
App. country/citz.	Yes	Yes	Yes	Yes	Yes	Yes
N	4,842	4,797	3,865	3,787	2,663	2,612
R^2	0.090	0.091	0.093	0.093	0.088	0.090
Within-R^2	0.000	0.000	0.001	0.001	0.001	0.002
Mean of Y	0.452	0.452	0.431	0.432	0.449	0.448
SD of Y	0.498	0.498	0.495	0.495	0.497	0.497

Dependent variables are indicators for funding offered. Parentheses report two-sided P-values from t-tests of the null hypothesis that the coefficient equals zero, calculated using standard errors clustered at the panel-scheme-year level. The second block lists the included fixed effects. STG: Starting Grant. COG: Consolidator Grant. ADG: Advanced Grant.

Supplementary Table 29 Same-country and same-citizenship homophily in panel decisions by domain in Step 1.

	PE		LS		SH	
	Assigned A					
	(1)	(2)	(3)	(4)	(5)	(6)
Share of same country/ same citz. panelists	0.328		0.007		0.436	
	(0.006)		(0.954)		(0.003)	
	[0.095, 0.561]		[-0.231, 0.245]		[0.148, 0.724]	
Share of same country/ diff. citz. panelists	0.100		0.189		0.407	
	(0.455)		(0.207)		(0.007)	
	[-0.164, 0.365]		[-0.106, 0.483]		[0.115, 0.699]	
Share of diff. country/ same citz. panelists	0.092		0.012		0.241	
	(0.297)		(0.928)		(0.052)	
	[-0.082, 0.266]		[-0.253, 0.277]		[-0.002, 0.483]	
Same country/ same citz. chair		0.002		0.054		0.091
		(0.941)		(0.074)		(0.009)
		[-0.042, 0.045]		[-0.005, 0.113]		[0.023, 0.159]
Same country/ diff. citz. chair		0.017		0.017		0.024
		(0.469)		(0.491)		(0.311)
		[-0.028, 0.061]		[-0.031, 0.065]		[-0.023, 0.070]
Diff. country/ same citz. chair		0.038		-0.021		0.047
		(0.069)		(0.386)		(0.020)
		[-0.003, 0.079]		[-0.069, 0.027]		[0.007, 0.086]
Panel-Scheme-Year	Yes	Yes	Yes	Yes	Yes	Yes
App. country/citz.	Yes	Yes	Yes	Yes	Yes	Yes
N	15,658	15,446	10,238	10,238	10,304	10,237
R^2	0.094	0.095	0.121	0.121	0.083	0.083
Within-R^2	0.001	0.000	0.000	0.001	0.002	0.001
Mean of Y	0.344	0.343	0.335	0.335	0.297	0.297
SD of Y	0.475	0.475	0.472	0.472	0.457	0.457

Dependent variable is an indicator for receiving an A. Parentheses report two-sided P-values from t-tests of the null hypothesis that the coefficient equals zero, calculated using standard errors clustered at the panel-scheme-year level. The second block lists the included fixed effects. PE: Physical Sciences and Engineering. LS: Life Sciences. SH: Social Sciences and Humanities.

Supplementary Table 30 Same-country and same-citizenship homophily in panel decisions (assigned A) by domain in Step 2.

	PE		LS		SH	
	Assigned A					
	(1)	(2)	(3)	(4)	(5)	(6)
Share of same country/ same citz. panelists	-0.026		0.331		0.110	
	(0.889)		(0.125)		(0.749)	
	[-0.398, 0.345]		[-0.093, 0.754]		[-0.568, 0.787]	
Share of same country/ diff. citz. panelists	-0.092		0.158		0.068	
	(0.612)		(0.453)		(0.823)	
	[-0.449, 0.265]		[-0.256, 0.572]		[-0.538, 0.675]	
Share of diff. country/ same citz. panelists	0.130		0.069		0.169	
	(0.390)		(0.700)		(0.455)	
	[-0.167, 0.427]		[-0.284, 0.422]		[-0.278, 0.615]	
Same country/ same citz. chair		0.014		0.003		0.097
		(0.648)		(0.933)		(0.067)
		[-0.047, 0.076]		[-0.078, 0.085]		[-0.007, 0.201]
Same country/ diff. citz. chair		0.005		-0.011		0.068
		(0.895)		(0.791)		(0.100)
		[-0.067, 0.076]		[-0.097, 0.074]		[-0.013, 0.149]
Diff. country/ same citz. chair		-0.057		0.161		0.018
		(0.088)		(<0.001)		(0.688)
		[-0.123, 0.009]		[0.076, 0.246]		[-0.070, 0.106]
Panel-Scheme-Year	Yes	Yes	Yes	Yes	Yes	Yes
App. country/citz.	Yes	Yes	Yes	Yes	Yes	Yes
N	5,185	5,033	3,272	3,272	2,922	2,899
R^2	0.194	0.196	0.180	0.183	0.116	0.118
Within-R^2	0.000	0.001	0.001	0.004	0.000	0.002
Mean of Y	0.685	0.685	0.659	0.659	0.521	0.521
SD of Y	0.464	0.464	0.474	0.474	0.500	0.500

Dependent variables are indicators for receiving an A. Parentheses report two-sided P-values from t-tests of the null hypothesis that the coefficient equals zero, calculated using standard errors clustered at the panel-scheme-year level. The second block lists the included fixed effects. PE: Physical Sciences and Engineering. LS: Life Sciences. SH: Social Sciences and Humanities.

Supplementary Table 31 Same-country and same-citizenship homophily in panel decisions (funding offered) by domain in Step 2.

	PE		LS		SH	
	Funding Offered					
	(1)	(2)	(3)	(4)	(5)	(6)
Share of same country/ same citz. panelists	-0.050		0.335		0.172	
	(0.829)		(0.201)		(0.581)	
	[-0.504, 0.404]		[-0.181, 0.850]		[-0.444, 0.788]	
Share of same country/ diff. citz. panelists	-0.315		-0.153		-0.049	
	(0.134)		(0.546)		(0.880)	
	[-0.729, 0.098]		[-0.652, 0.346]		[-0.693, 0.594]	
Share of diff. country/ same citz. panelists	0.216		0.190		0.062	
	(0.232)		(0.307)		(0.800)	
	[-0.140, 0.572]		[-0.176, 0.556]		[-0.423, 0.547]	
Same country/ same citz. chair		0.012		-0.018		0.107
		(0.759)		(0.688)		(0.065)
		[-0.065, 0.089]		[-0.104, 0.069]		[-0.007, 0.221]
Same country/ diff. citz. chair		0.054		0.007		0.023
		(0.135)		(0.888)		(0.608)
		[-0.017, 0.125]		[-0.096, 0.111]		[-0.066, 0.111]
Diff. country/ same citz. chair		-0.013		0.114		-0.008
		(0.771)		(0.024)		(0.853)
		[-0.100, 0.074]		[0.015, 0.212]		[-0.098, 0.082]
Panel-Scheme-Year	Yes	Yes	Yes	Yes	Yes	Yes
App. country/citz.	Yes	Yes	Yes	Yes	Yes	Yes
N	5,185	5,033	3,272	3,272	2,922	2,899
R^2	0.096	0.098	0.101	0.101	0.089	0.090
Within-R^2	0.001	0.000	0.001	0.002	0.000	0.001
Mean of Y	0.425	0.426	0.454	0.454	0.469	0.469
SD of Y	0.494	0.494	0.498	0.498	0.499	0.499

Dependent variables are indicators for funding offered. Parentheses report two-sided P-values from t-tests of the null hypothesis that the coefficient equals zero, calculated using standard errors clustered at the panel-scheme-year level. The second block lists the included fixed effects. PE: Physical Sciences and Engineering. LS: Life Sciences. SH: Social Sciences and Humanities.

Supplementary Table 32 Same-country and same-citizenship homophily in panel decisions measured by reviewers only.

	Step 1	Step 2	
	Assigned A		Funding Offered
	(1)	(2)	(3)
Share of same country/ same citz. reviewers	0.080	0.153	0.132
	(0.012)	(0.033)	(0.091)
	(0.018, 0.143)	(0.012, 0.293)	(-0.021, 0.286)
Share of same country/ diff. citz. reviewers	-0.035	0.012	-0.002
	(0.283)	(0.873)	(0.974)
	(-0.099, 0.029)	(-0.132, 0.156)	(-0.145, 0.141)
Share of diff. country/ same citz. reviewers	0.060	-0.014	0.039
	(0.062)	(0.818)	(0.543)
	(-0.003, 0.123)	(-0.130, 0.103)	(-0.087, 0.166)
Panel-Scheme-Year	Yes	Yes	Yes
App. country/citz.	Yes	Yes	Yes
N	36,670	11,645	11,645
R^2	0.078	0.159	0.070
Within-R^2	0.000	0.000	0.000
Mean of Y	0.326	0.634	0.445
SD of Y	0.469	0.482	0.497

Dependent variables are indicators for receiving an A and for funding offered. Parentheses report two-sided P-values from t-tests of the null hypothesis that the coefficient equals zero, calculated using standard errors clustered at the panel-scheme-year level. The second block lists the included fixed effects.

Supplementary Table 33 Same-country and same-citizenship homophily in panel decisions: Average marginal effects from Logit regressions.

	Step 1		Step 2			
	Assigned A				Funding Offered	
	(1)	(2)	(3)	(4)	(5)	(6)
Share of same country/ same citz. panelists	0.264		0.164		0.135	
	(<0.001)		(0.195)		(0.348)	
	[0.124, 0.404]		[-0.084, 0.412]		[-0.147, 0.417]	
Share of same country/ diff. citz. panelists	0.264		0.115		-0.099	
	(0.001)		(0.346)		(0.461)	
	[0.114, 0.415]		[-0.125, 0.355]		[-0.363, 0.164]	
Share of diff. country/ same citz. panelists	0.155		0.154		0.193	
	(0.009)		(0.119)		(0.069)	
	[0.038, 0.271]		[-0.040, 0.347]		[-0.015, 0.402]	
Same country/ same citz. chair		0.029		0.021		0.016
		(0.048)		(0.368)		(0.511)
		[0.000, 0.058]		[-0.025, 0.068]		[-0.032, 0.064]
Same country/ diff. citz. chair		0.019		0.013		0.039
		(0.145)		(0.565)		(0.092)
		[-0.007, 0.045]		[-0.031, 0.057]		[-0.006, 0.084]
Diff. country/ same citz. chair		0.027		0.016		0.016
		(0.017)		(0.492)		(0.549)
		[0.005, 0.050]		[-0.029, 0.061]		[-0.035, 0.066]

Dependent variables are indicators for receiving an A and for funding offered. Reported estimates are average marginal effects from logit regressions. Parentheses report two-sided P-values from t-tests of the null hypothesis that the coefficient equals zero, calculated using standard errors clustered at the panel-scheme-year level. The second block lists the included fixed effects.
