# Supplementary material for: Homophily in grant evaluations
Source: Nat Hum Behav. 2026 Aug 13;10(10):1943–52. doi: 10.1038/s41562-026-02554-w (PMC13683047; doi:10.1038/s41562-026-02554-w)
Supplement: Supplementary file 2 [file 41562_2026_2554_MOESM2_ESM.pdf]

nature portfolio

Corresponding author(s): Felix Holub

Last updated by author(s): Jun 29, 2026

Reporting Summary

Nature Portfolio wishes to improve the reproducibility of the work that we publish. This form provides structure for consistency and transparency in reporting. For further information on Nature Portfolio policies, see our Editorial Policies and the Editorial Policy Checklist.

Statistics

For all statistical analyses, confirm that the following items are present in the figure legend, table legend, main text, or Methods section.

n/a	Confirmed	
☐	☒	The exact sample size (*n*) for each experimental group/condition, given as a discrete number and unit of measurement
☒	☐	A statement on whether measurements were taken from distinct samples or whether the same sample was measured repeatedly
☐	☒	The statistical test(s) used AND whether they are one- or two-sided<br>*Only common tests should be described solely by name; describe more complex techniques in the Methods section.*
☐	☒	A description of all covariates tested
☐	☒	A description of any assumptions or corrections, such as tests of normality and adjustment for multiple comparisons
☐	☒	A full description of the statistical parameters including central tendency (e.g. means) or other basic estimates (e.g. regression coefficient) AND variation (e.g. standard deviation) or associated estimates of uncertainty (e.g. confidence intervals)
☐	☒	For null hypothesis testing, the test statistic (e.g. *F*, *t*, *r*) with confidence intervals, effect sizes, degrees of freedom and *P* value noted<br>*Give P values as exact values whenever suitable.*
☒	☐	For Bayesian analysis, information on the choice of priors and Markov chain Monte Carlo settings
☒	☐	For hierarchical and complex designs, identification of the appropriate level for tests and full reporting of outcomes
☐	☒	Estimates of effect sizes (e.g. Cohen's *d*, Pearson's *r*), indicating how they were calculated

Our web collection on statistics for biologists contains articles on many of the points above.

Software and code

Policy information about availability of computer code

Data collection	The data were provided by the ERCEA. Additional data were obtained from the OpenAlex (V1) API using Python 3.12.4 with pyalex 0.15.1.
Data analysis	The data were analysed in Julia 1.10.5. and models were estimated with FixedEffectModels.jl 1.11.0. Embedding were obtained using Python 3.12.4 with the SPECTER2 model (allenai/specter2_aug2023refresh_base) and proximity adapter (allenai/specter2_aug2023refresh), accessed through Hugging Face using transformers 4.43.4 and adapters 1.0.0. Code used for data processing, analysis and figure generation is available at https://doi.org/10.6084/m9.figshare.30789395. The repository includes pseudo data that allow the code to be run without access to the confidential ERCEA data.

For manuscripts utilizing custom algorithms or software that are central to the research but not yet described in published literature, software must be made available to editors and reviewers. We strongly encourage code deposition in a community repository (e.g. GitHub). See the Nature Portfolio guidelines for submitting code & software for further information.

Data

Policy information about availability of data

All manuscripts must include a data availability statement. This statement should provide the following information, where applicable:

- Accession codes, unique identifiers, or web links for publicly available datasets
- A description of any restrictions on data availability
- For clinical datasets or third party data, please ensure that the statement adheres to our policy

This research is based on data provided by the European Research Council Executive Agency (ERCEA) and is accessed exclusively within the framework of the expert contract for this assignment. In accordance with the contract's terms and the ERCEA's instructions, the use and disclosure of these data are subject to confidentiality obligations. As a result, the underlying data cannot be made publicly available. Any reproduction of the analyses by independent researchers would require direct authorization from the ERCEA and compliance with their data access procedures. The authors' access to the data was strictly limited to the scope of their expert assignment.Researchers interested in accessing the data would need to contact ERCEA unit ERCEA.B.1 (Head: Raluca Ionescu, Contact: https://op.europa.eu/s/AiAG). Access is at ERCEA's discretion and is not controlled by the authors.

Research involving human participants, their data, or biological material

Policy information about studies with human participants or human data. See also policy information about sex, gender (identity/presentation), and sexual orientation and race, ethnicity and racism.

Reporting on sex and gender	All data was already obtained by the ERCEA. The ERCEA collect information on self-reported gender.
Reporting on race, ethnicity, or other socially relevant groupings	*Please specify the socially constructed or socially relevant categorization variable(s) used in your manuscript and explain why they were used. Please note that such variables should not be used as proxies for other socially constructed/relevant variables (for example, race or ethnicity should not be used as a proxy for socioeconomic status).* *Provide clear definitions of the relevant terms used, how they were provided (by the participants/respondents, the researchers, or third parties), and the method(s) used to classify people into the different categories (e.g. self-report, census or administrative data, social media data, etc.)* *Please provide details about how you controlled for confounding variables in your analyses.*
Population characteristics	See below.
Recruitment	No participants were recruited for this study.
Ethics oversight	No additional institutional ethics oversight was sought, as the analysis used existing administrative data under an ERCEA expert contract.

Note that full information on the approval of the study protocol must also be provided in the manuscript.

Field-specific reporting

Please select the one below that is the best fit for your research. If you are not sure, read the appropriate sections before making your selection.

☐ Life sciences ☒ Behavioural & social sciences ☐ Ecological, evolutionary & environmental sciences

For a reference copy of the document with all sections, see nature.com/documents/nr-reporting-summary-flat.pdf

Behavioural & social sciences study design

All studies must disclose on these points even when the disclosure is negative.

Study description	This quantitative study investigates homophily in grant evaluation processes using administrative data from the European Research Council (ERC). We employ econometric methods to causally identify the effects of shared gender, citizenship, and country of work between applicants and reviewers on individual grant scores, and to correlationally assess their impact on panel-level funding decisions.
Research sample	The research sample comprises 37,073 ERC grant applications covering all ERC scientific domains and funding schemes included in the administrative data. The dataset includes individual reviewer scores, panel decisions, and demographic or affiliation information for applicants and reviewers, including gender, citizenship and country of work. Age was not available in the data. The sample represents the population of ERC applications covered by the available administrative records and eligible for the analyses, subject to variable availability; it is not intended to be representative of the broader population of scientists or research grant applicants. The study sample was chosen because it constitutes the relevant administrative population for analysing reviewer–applicant and panel–applicant matches in ERC evaluations.
Sampling strategy	This study uses a census of all available ERC grant applications and associated reviewer data from 2019 to 2023. No sampling strategy was applied; all eligible observations within this period were included, subject to variable availability for each analysis.

Data collection	Data were collected by the European Research Council Executive Agency (ERCEA) as part of its standard grant evaluation and administrative processes. The records were generated through ERCEA's administrative grant-management and evaluation systems, including application records, reviewer assignments, reviewer scores and panel-decision records. The authors accessed these pre-existing administrative records from ERCEA under the terms of a confidential expert contract. No primary data collection was performed by the authors, and the authors did not interact with applicants, reviewers or panel members. Questions about the presence of researchers or other individuals during data collection are therefore not applicable. Blinding to experimental condition is also not applicable because this is an observational study with no experimental intervention or treatment assignment.
Timing	The data analysed cover ERC grant application cycles from 2019 to 2023. Data were generated by ERCEA during the regular administrative and evaluation processes for these cycles. There were no separate experimental collection waves or cohort-specific collection periods.
Data exclusions	No observations were excluded based on outcomes, estimated effects or outlier status. Analyses used all eligible administrative records with non-missing variables required for the corresponding specification. Exclusions therefore arise only from pre-specified data requirements, such as missing demographic or affiliation information or missing variables needed to construct academic-closeness, coauthorship-network or topic-match measures. Exact sample sizes for each analysis are reported in the corresponding figure legends and Supplementary Tables.
Non-participation	As this study uses pre-existing administrative data, there was no active recruitment and no active non-participation by individuals.
Randomization	This observational study does not involve experimental randomization.

Reporting for specific materials, systems and methods

We require information from authors about some types of materials, experimental systems and methods used in many studies. Here, indicate whether each material, system or method listed is relevant to your study. If you are not sure if a list item applies to your research, read the appropriate section before selecting a response.

Materials & experimental systems

n/a	Involved in the study
☒	☐ Antibodies
☒	☐ Eukaryotic cell lines
☒	☐ Palaeontology and archaeology
☒	☐ Animals and other organisms
☒	☐ Clinical data
☒	☐ Dual use research of concern
☒	☐ Plants

Methods

n/a	Involved in the study
☒	☐ ChIP-seq
☒	☐ Flow cytometry
☒	☐ MRI-based neuroimaging

Plants

Seed stocks	*Report on the source of all seed stocks or other plant material used. If applicable, state the seed stock centre and catalogue number. If plant specimens were collected from the field, describe the collection location, date and sampling procedures.*
Novel plant genotypes	*Describe the methods by which all novel plant genotypes were produced. This includes those generated by transgenic approaches, gene editing, chemical/radiation-based mutagenesis and hybridization. For transgenic lines, describe the transformation method, the number of independent lines analyzed and the generation upon which experiments were performed. For gene-edited lines, describe the editor used, the endogenous sequence targeted for editing, the targeting guide RNA sequence (if applicable) and how the editor was applied.*
Authentication	*Describe any authentication procedures for each seed stock used or novel genotype generated. Describe any experiments used to assess the effect of a mutation and, where applicable, how potential secondary effects (e.g. second site T-DNA insertions, mosiacism, off-target gene editing) were examined.*
