# Supplementary material for: Homophily in grant evaluations
Source: Nat Hum Behav. 2026 Aug 13;10(10):1943–52. doi: 10.1038/s41562-026-02554-w (PMC13683047; doi:10.1038/s41562-026-02554-w)
Supplement: Supplementary file 3 [file 41562_2026_2554_MOESM3_ESM.pdf]

nature portfolio

Peer Review File

Homophily in Grant Evaluations

Corresponding Author: Dr Felix Holub

Version 0:

Decision Letter:

21st January 2026

Dear Dr Holub,

Thank you once again for your manuscript, entitled "Homophily in Grant Evaluations", and for your patience during the peer review process.

Your Article has now been evaluated by 3 referees. You will see from their comments copied below that, although they find your work of considerable potential interest, they have raised quite substantial concerns. In light of these comments, we cannot accept the manuscript for publication in the current form, but would be interested in considering a revised version if you are willing and able to fully address reviewer and editorial concerns.

We are committed to providing a fair and constructive peer-review process. Do not hesitate to contact us if there are specific requests from the reviewers that you believe are technically impossible or unlikely to yield a meaningful outcome.

In particular, we feel that it will be necessary to either considerably strengthen, or remove, the analyses of mechanisms. Reviewers #1 and #3 both comment on the mechanisms section as currently being less convincing than the rest of the study. You should either strengthen this section along the lines that Reviewer #3 recommends, or if this is not possible, remove it. You should also carry out all of the other changes and additional analyses suggested by the reviewers. We feel that a stronger Discussion section will greatly improve the work.

Finally, your revised manuscript must comply fully with our editorial policies and formatting requirements. Failure to do so will result in your manuscript being returned to you, which will delay its consideration. To assist you in this process, I have attached a checklist that lists all of our requirements. If you have any questions about any of our policies or formatting, please don't hesitate to contact me.

If you wish to submit a suitably revised manuscript, we would hope to receive it within 4 months. I would be grateful if you could contact us as soon as possible if you foresee difficulties with meeting this target resubmission date.

With your revision, please:

• Include a “Response to the editors and reviewers” document detailing, point-by-point, how you addressed each editor and referee comment. If no action was taken to address a point, you must provide a compelling argument. When formatting this document, please respond to each reviewer comment individually, including the full text of the reviewer comment verbatim followed by your response to the individual point. This response will be used by the editors to evaluate your revision and sent back to the reviewers along with the revised manuscript.

• Highlight all changes made to your manuscript or provide us with a version that tracks changes.

Please use the link below to submit your revised manuscript and related files:

Link Redacted

Note: This URL links to your confidential home page and associated information about manuscripts you may have submitted, or that you are reviewing for us. If you wish to forward this email to co-authors, please delete the link to your homepage.

Thank you for the opportunity to review your work. Please do not hesitate to contact me if you have any questions or would like to discuss the required revisions further.

Sincerely,

[redacted]

[redacted]
Nature Human Behaviour

REVIEWER COMMENTS:

Reviewer #1 (Remarks to the Author):

This paper examines whether grant reviewers favor applicants who share their gender, citizenship, or country of work ("homophily") in research. They find small but statistically significant gender homophily and larger effects for shared citizenship and country of work. Reviewers who share these national attributes with applicants award higher scores to the same proposal, especially when both country and citizenship align.

At the panel level, applicants are more likely to advance past the first evaluation stage—and ultimately to be funded—when more panel members or the chair share their country or citizenship. The paper concludes that homophily, particularly along national lines, challenges the objectivity of grant evaluation and has different consequences across countries.

This work has several strengths:

1. The analysis is based on 37,073 proposals submitted to a prominent and important European funding scheme, the European Research Council (ERC).

2. The authors address different types of homophily, including same gender, same country, same citizenship, and same rank.

3. They exploit within-proposal and within-reviewer variation to causally separate reviewer bias from (unobserved) proposal quality.

4. The analyses distinguish between the assessment of the PI and the project.

5. The statistical methods are state of the art.

The work is timely, original and important and has the potential to reduce bias and make research funding fairer. However, there are also some weaknesses:

1. The analysis of panel-level homophily is based on the share of panel members who match the applicant's demographic characteristics. However, only a small number of assigned panel members drive the discussion, ranking, and funding decisions, while other panel members - who have not studied the proposal in detail - tend to defer to them. It would therefore be more informative to focus on assigned reviewers rather than the full panel.

2. The authors use semantic similarity measures and co-authorship networks to capture intellectual proximity, but they do not test "same (sub-)discipline" as a standalone homophily dimension, that is, whether reviewers favour proposals that are closer to their own field per se. In my experience, panel composition and epistemic matching can substantially influence evaluations. Prior studies of grant review show that reviewers with greater topic-specific expertise evaluate applications differently, implying that closer topical fit affects scoring (1).

3. I was not convinced by the analyses aimed at identifying the channels through which homophily arises (e.g. strategic motives, normative motives, commonality of taste). In my view, these mechanisms cannot be reliably identified using observational data of this kind. As a result, this section of the manuscript appears speculative and comparatively weak.

4. The ERC employs a two-stage evaluation process, and at the second stage four or more external peer reviewers provide written assessments that inform the final funding decision. These external reviews are influential but are not considered in the analysis. Evidence from the Swiss National Science Foundation shows gender-specific homophily in external peer-review scores (2). At a minimum, the role of external reviewers and their potential contribution to homophily should be discussed.

5. The authors note that not all A-rated proposals can be funded and that proposals are therefore ranked, but they do not explain how this ranking is performed in practice. Ranking is often problematic, as panel members may introduce additional, informal criteria, not specified in the regulations, to justify higher or lower ranks.

6. Surprisingly, the paper does not include a dedicated Discussion section, but only relatively brief conclusions. The authors should add a Discussion that:

- explicitly reflects on the strengths and weaknesses of the study,
- situates the findings in relation to the growing literature on bias and influence in grant (and possibly journal) peer review (1, 3–8),
- discusses the meaning and implications of the results, and
- outlines concrete directions for future research.

7. Relatedly, the paper does not sufficiently discuss policy interventions that could reduce or eliminate homophily bias. The only proposal advanced - excluding reviewers who share the applicant's citizenship or country of work - seems impractical and unrealistic. Other options deserve consideration, including:

-Blinding panel members or reviewers to applicant demographics, at least at the first evaluation stage;
- Partial lotteries for proposals that are clearly fundable but not outstanding, which are especially vulnerable to biased marginal decisions;
- More explicit and tightly defined evaluation criteria, as well as greater diversity of panels in terms of gender, career stage, and background.

8. Finally, I disagree with the authors' claim that "while the ERC is international by design, similar dynamics are likely to arise in national funding agencies." In national funding systems, panel members and reviewers are often competitors of the applicants for scarce funding. In such contexts, homophily may instead lead to harsher, not more favourable, assessments.

In conclusion, this is a methodologically rigorous and substantively important paper that advances the literature on bias in research evaluation. Its main limitations concern panel-level causality, the identification of underlying mechanisms, and the lack of a well-developed Discussion that situates the findings within the broader literature and policy context.

1. Gallo SA et al. The Influence of Peer Reviewer Expertise on the Evaluation of Research Funding Applications. PLOS ONE. 2016 Oct 21;11(10):e0165147.
2. Severin A et al. Gender and other potential biases in peer review: cross-sectional analysis of 38 250 external peer review reports. BMJ Open. 2020 Aug 1;10(8):e035058.
3. Heyard R, et al. Rethinking the Funding Line at the Swiss National Science Foundation: Bayesian Ranking and Lottery. Stat Public Policy. 2022 Dec 31;9(1):110–21.
4. Horbach SPJM et al. Partial lottery can make grant allocation more fair, more efficient, and more diverse. Sci Public Policy. 2022 Aug 17;49(4):580–2.
5. Schmaling KB, Gallo SA. An experimental study of simulated grant peer review: Gender differences and psychometric characteristics of proposal scores. PLoS One. 2024 Dec 17;19(12):e0315567.
6. Oxley K. Partial and particularistic: grant peer review panels evaluating interdisciplinarity. Res Evaluat. 2025;34:rvaf037.
7. Madsen EB, Mongeon P, Schneider JW. Gender disparity in funding rates in double-blind grant peer review: The case of the Villum Experiment. Quant Sci Stud. 2025 Aug 18;6:774–95.
8. Sims Gould J et al. Threats to grant peer review: a qualitative study. BMJ Open. 2025 Feb 20;15(2):e091666.

Reviewer #2 (Remarks to the Author):

This manuscript contributes to a growing set of studies documenting the ways in which the peer-review system in science is biased, in this particular case as it relates to the review of grant proposals. The authors exploit the substantial nationality variation in the composition of review panels and applicant pool within the ERC (European Research Council) grant program to assess in-group (homophily) bias of reviewers attributable to nationality (as well as gender). Interestingly, while there has been notable work on gender bias in science - relative to their observed effect attributable to gender homophily, this study finds within-group nationality bias to be substantially larger in magnitude. As such, this study provides a grounded estimation that, while not necessarily unimaginable, should certainly raise alarms and can be the impetus for policy changes to address the role of in-group bias in grant reviews (and publication review) for funding bodies (international journals) the world over.

The manuscript exploits unit-level ERC grant application and review panel data, and appropriately highlights the opportunities (comprehensive data) and challenges (e.g. selection effects) associated with identifying causal effects that are offered by this valuable data sample, in particular by way of a research design that leverages within-proposal and within-reviewer variation. The motivation and methodological description are effective and concise, the contributions to the literature are clearly stated and supported, a number of alternative mechanisms are explored, and overall the manuscript is organized and reads very well.

To be balanced, the main critique is that the effect sizes are, on average, not terribly large in relation to the observed variation in scores among winners, ie the in-group bias is not sufficiently large to be entirely deterministic of outcomes. To some degree, this does provide some sense of relief. Nevertheless, I do recommend publication of this important study based upon its elegance, depth of exploration, and consistency of results.

Below are a few comments for the authors to consider.

Abstract: The reference to “normative” concerns is vague, and if there is sufficient room for elaboration than this adjective should be elaborated so that it is clear what aspect of norms are being referred to, ie according to its clear definition on page 8.

Figure 3: There is a typo in the panel labels, which are in the order a,c,b,d from top to bottom.

Figure 4: Recommend modifying the label “Share different” to “Share of different” to be consistent with text, and also to improve the clarity of the meaning, as without the “of” the label could be interpreted very differently if a reader only were to read the figures.

Panel decisions: It should be made more clear in the text that y_i is an (0,1) variable; in which case, it’s not clear why a logit or similar binary outcome model was used?

Discussion: it would be instructive to more specifically discuss recommendations/reflections regarding the multi-stage format of the ERC system. What are the pros/cons of this format associated with the findings; and what steps could be made to improve the fidelity of the peer review system – e.g. excluding same/country-citizenship matches among reviewer in Step 1, at the cost of reducing the sample size for determining the Step 1 proposal grade.

Reviewer #2 (Remarks on code availability):

I am not familiar with the code-base being used, and so am unable to assess it. In principle the data structure is basic, the observations are well-defined, and the methodology straightforward - and so I do not expect any issues with the provided code.

Reviewer #3 (Remarks to the Author):

Summary: The paper examines whether homophily between applicants and evaluators, along the dimensions of gender, country of employment, and citizenship, influences evaluations and funding decisions for European Research Council (ERC) Starting, Consolidator, and Advanced Grants. Using a causal identification strategy, the authors show that shared characteristics along all three dimensions increase the likelihood of receiving a high rating from evaluators. However, the effect of gender homophily is economically negligible, whereas homophily in country of employment and citizenship has larger effects. The authors argue that the most likely drivers of more favorable reviews for same country/same citizenship applicants are strategic (reciprocity within a group) or normative (stronger in-group loyalty) motives. For the panel decisions, the results are correlational. A higher share of women on the panel lowers the odds of women receiving funding, while a higher fraction of panel members with the same citizenship/country leads to a higher evaluation at the first step, but has no effect on the panel’s decision in the second step, in particular funding offered.

Evalution: I find much to like about this paper. ERC grants are important sources of funding and therefore it is worth investigating whether homophily is shaping the decisions of evaluators and panels, which may indicate a bias.
I therefore do believe that the paper has the potential to make a substantial Applied/Societal-/Policy-related advance. However, there are some shortcomings regarding the exposition, the suggested mechanisms and the analysis conducted.

• I do not find the mechanisms explaining why evaluators rate academics working in their own country or with the same citizenship more highly convincing. First, a combination of factors such as information, reciprocity, or in-group loyalty might be at play. Such an interplay cannot be ruled out. Moreover, at the panel level, homophily appears only in step one of the evaluation process, but not in the second. What drives this difference between the panel and evaluators? And why does a homophily bias emerge in the panel at step 1, but not in step 2? How does this align with the proposed mechanisms? The suggested mechanisms also raise the question of why these considerations do not seem to apply to women. Overall, I find the discussion of mechanisms raises more questions than it answers, and as it is only addressed for evaluators, it does not seem very meaningful. However, I also don’t think much can be done to clarify the mechanisms with the data available. Overall, I would not mind if the authors removed the discussion of mechanisms altogether. If this is not the preferred option, I would suggest (i) providing a discussion of mechanisms related to homophily for gender and country/citizenship, (ii) explaining why differences exist between the two categories– gender and country/citizenship, and (iii) highlighting why we see disparities in homophily between evaluators, panel 1, and panel 2.

• I find the discussion of same-country and same-citizenship effects in panel decisions underdeveloped. First, for step 1 and step 2, no tables are referenced, making it challenging to evaluate what is going on. Which tables show these effects?

• The part I found most unclear was the discussion of homophily-affected selection in step 2. The argument is that, given homophily in previous stages, homophily may ultimately still affect the funding decision even though, at the step 2 panel, there is no homophily present. The authors therefore calculate the probability of receiving funding conditional on reaching Step 2, multiplied by the probability of reaching Step 2. They then take the derivative and obtain a term in the methodology section, but it is not clear where this term comes from. This needs to be explained much better in the methodology section. And where do the numbers in the main section come from regarding the probabilities come from? Can they be matched to a table?

• Economic significance: What is the overall economic significance of homophily for the probability of receiving funding? There should be a discussion of how large these effects are. At this stage, it is not obvious that there is a bias in the ultimate funding decision or how economically meaningful it is. It would also be useful to understand how the probability of funding differs for applicants from a large country versus a small country (for example, Germany versus Luxembourg), to gain some intuition.

Reviewer #3 (Remarks on code availability):

The data is proprietary, the authors cannot share it, so just reviewing the code is meaningless.

Version 1:

Decision Letter:

Our ref: NATHUMBEHAV-25115896A

3rd June 2026

Dear Dr. Holub,

Thank you for submitting your revised manuscript "Homophily in Grant Evaluations" (NATHUMBEHAV-25115896A). It has now been seen by the original referees and their comments are below. As you can see, the reviewers find that the paper has improved in revision. We will therefore be happy in principle to publish it in Nature Human Behaviour, pending minor revisions to satisfy the referees' final requests and to comply with our editorial and formatting guidelines.

We are now performing detailed checks on your paper and will send you a checklist detailing our editorial and formatting requirements within two weeks. Please do not upload the final materials and make any revisions until you receive this additional information from us.

Please do not hesitate to contact me if you have any questions.

Sincerely,
[redacted]

[redacted]
Nature Human Behaviour

Reviewer #1 (Remarks to the Author):

The authors have addressed my main concerns: they added assigned-reviewer analyses, introduced a topic-match control, toned down speculative mechanism claims, clarified the role of external referees, and expanded the Discussion and policy implications.

Two minor points:

1. The authors now present new results in the Discussion section and I wondered whether they should be moved to the Results.

2. They use the term 'no evidence' but a p value >0.05 does not mean there is no evidence. Suggest you rephrase, for example 'little evidence' or 'no strong evidence'.

Reviewer #2 (Remarks to the Author):

Thank you for addressing my initial set of comments in full.

While I follow your argument that using a linear probability model facilitates consistent comparison with the other models based upon numerical scores, it will still not sit well with those familiar with the potential pitfalls of applying linear models to

binary dependent variables. Hence, my only remaining recommendation is to include the Logit model results in the supplementary information.

Reviewer #2 (Remarks on code availability):

Falling short of running everything locally, the code deposition looks sufficiently complete so as to provide a solid avenue for the replication of the results.

Reviewer #3 (Remarks to the Author):

Thanks for addressing all my comments. I have two very minor comments left, which are merely cosmetic.

1) you emphasize that your contribution is to provide a causal estimate of homophily. This is correct for the evaluation in the first stage of the ERC process, but not for the overall success rate. It would be good to be precise on this.

2) p.11 lines 266-268: it would be good to state in one short sentence how the results of the robustness check compare.

Open Access This Peer Review File is licensed under a Creative Commons Attribution 4.0 International License, which permits use, sharing, adaptation, distribution and reproduction in any medium or format, as long as you give appropriate credit to the original author(s) and the source, provide a link to the Creative Commons license, and indicate if changes were made.
In cases where reviewers are anonymous, credit should be given to 'Anonymous Referee' and the source.
The images or other third party material in this Peer Review File are included in the article's Creative Commons license, unless indicated otherwise in a credit line to the material. If material is not included in the article's Creative Commons license and your intended use is not permitted by statutory regulation or exceeds the permitted use, you will need to obtain permission directly from the copyright holder.
To view a copy of this license, visit https://creativecommons.org/licenses/by/4.0/

Response Letter Reviewer 1

We thank the reviewer for the detailed and constructive report and for highlighting both the strengths and the key limitations of our initial submission. We address each suggestion in turn below.

1. **The analysis of panel-level homophily is based on the share of panel members who match the applicant's demographic characteristics. However, only a small number of assigned panel members drive the discussion, ranking, and funding decisions, while other panel members - who have not studied the proposal in detail - tend to defer to them. It would therefore be more informative to focus on assigned reviewers rather than the full panel.**

We agree that the panel members assigned to prepare the individual assessments are likely to be particularly influential in the subsequent discussion. (In our analysis of individual reviews, we focus on these assigned reviewers and document homophily in their numeric scores using proposal and reviewer fixed effects.)

Your comment pertains to our panel-outcome regressions, where our baseline exposure measure is the share of all panel members (and separately the chair) who match the applicant on country of work and/or citizenship. Following your suggestion, we re-estimated these panel-outcome regressions defining exposure using instead the share of assigned panel reviewers who match the applicant. We now report these alternative specifications in the Supplementary Information (Supplementary Tables 24–25) and add a pointer to them in the main text (p. 11).

The results show that the most robust positive association arises when applicant and evaluators match on both country of work and citizenship. Using assigned-reviewer shares, the coefficient on "same country/same citizenship" is positive and statistically significant for advancing in Step 1 (0.080, $p = 0.012$) and for receiving an A in Step 2 (0.153, $p = 0.033$), and positive for funding (0.132, $p = 0.091$). In contrast, the Step-1 associations for "same country only" and "same citizenship only" that are statistically significant using full-panel shares attenuate and become statistically insignificant when exposure is defined using assigned reviewers only. We interpret this pattern as reflecting that assigned-reviewer exposure is measured over a small number of reviewers per proposal (and thus has more limited variation), whereas full-panel measures capture the broader composition of the decision-making group in the panel meeting.

Finally, we note that any influence of the broader panel composition (beyond the assigned reviewers) is most plausible for proposals that are discussed most intensely in the meeting—namely borderline cases near the threshold for an A or for funding, and cases with diverging reviews. These proposals typically receive the most attention precisely because relative quality is difficult to assess and rank, which increases discretion in deliberation. These proposals also clearly necessitate a comparison and ranking between proposals that involved different panel members as evaluators, thereby including the entire panel in the discussion. This consideration motivates our focus on full-panel and chair composition in the main panel-outcome specifications.

2. **The authors use semantic similarity measures and co-authorship networks to capture intellectual proximity, but they do not test "same (sub-)discipline" as a standalone homophily dimension, that is, whether reviewers favour proposals that are closer to their own field per se. In my experience, panel composition and epistemic matching can substantially influence evaluations. Prior studies of grant review show that reviewers with greater topic-specific expertise evaluate applications differently, implying that closer topical fit affects scoring (1).**

We agree that reviewers' topic-specific expertise and the fit between a proposal and a reviewer's discipline can influence grant evaluations. While our data do not contain a direct subdiscipline

classification, we constructed a measure for topic proximity using ERC keywords and use it to assess whether our country- and citizenship-based match effects could be driven by epistemic matching.

Specifically, applicants select one or more ERC panel-specific keywords for their proposal. For each reviewer and year, we assign a "modal" ERC keyword defined as the keyword that most frequently appears among the proposals reviewed by that reviewer in that year (restricting to reviewer-years with at least 15 reviews to reduce noise). We then define a topic-match indicator equal to one if the reviewer's modal keyword is among the applicant's selected keywords (Methods: "Topic matches").

We include this topic-match indicator as an additional control in our within-proposal / within-reviewer regressions of individual scores. While in Step 1 the impact of having a topic match is statistically highly insignificant, in Step 2 panel members witch a matching topic award on average a 0.032 points ($p < 0.001$) higher project score and a 0.027 points ($p<0.001$) higher PI score. However, the estimated coefficients on same-country and same-citizenship matches are essentially unchanged relative to the baseline specification (Supplementary Table 14). This indicates that the estimated country- and citizenship-based homophily is unlikely to be explained by topical/epistemic matching as captured by the ERC keyword information.

3. **I was not convinced by the analyses aimed at identifying the channels through which homophily arises (e.g. strategic motives, normative motives, commonality of taste). In my view, these mechanisms cannot be reliably identified using observational data of this kind. As a result, this section of the manuscript appears speculative and comparatively weak.**

We revised the manuscript to substantially tone down and clarify the role of the "mechanism" analyses. In the previous version, our discussion could be read as attempting to pin down the underlying motives behind same-country/same-citizenship effects (e.g., strategic or normative channels), which is indeed not something our observational setting can cleanly establish.

Accordingly, we (i) removed the dedicated "mechanisms" section and any statements that attribute the effects to particular motives, and (ii) repositioned the remaining analyses as checks against two specific confounding explanations for the individual-score results: that the estimated match effects reflect omitted intellectual proximity ("shared tastes") or a pure informational-precision advantage. Concretely, we show that adding three proxies for topical/intellectual proximity, namely semantic similarity, coauthorship proximity, and ERC-keyword topic match, leaves the estimated country/citizenship match coefficients essentially unchanged (Fig. 2). We also examine the distribution of scores as a diagnostic for reviewer information as a confounder: if matches mainly increased reviewer information, one would expect more mass in both tails (Fig. 3). We now present these results cautiously as reducing the plausibility of these two confounding interpretations.

4. **The ERC employs a two-stage evaluation process, and at the second stage four or more external peer reviewers provide written assessments that inform the final funding decision. These external reviews are influential but are not considered in the analysis. Evidence from the Swiss National Science Foundation shows gender-specific homophily in external peer-review scores (2). At a minimum, the role of external reviewers and their potential contribution to homophily should be discussed.**

We agree that external (remote) reviewer reports are an important input into Step 2 deliberations and could themselves exhibit homophily. While our main analysis focuses on panel members, for this letter we additionally examined remote referees' recommendations where available in our data. For 2021-2023, remote referees provide a categorical recommendation ("not recommend / recommend / strongly recommend"); we therefore define an indicator for "strongly recommend" and estimate reviewer–applicant match effects on this binary outcome. The two tables below report the linear probability model results for (i) gender matches and (ii) country/citizenship matches (p-values and confidence bands in parentheses).

For same-gender matches, we find a small positive association in a specification with proposal fixed effects and reviewer-gender fixed effects (delta = 0.018, p = 0.019), but no association once we include remote-referee fixed effects (delta = -0.001, p = 0.978). For same-country and same-citizenship matches, we find positive and statistically significant associations both in a specification with reviewer country × citizenship fixed effects and when including remote-referee fixed effects, with the largest estimates for simultaneous matches in country and citizenship (delta = 0.169, $p < 0.001$ in the reviewer-fixed-effects specification).

We did not incorporate remote-referee analyses into the main text for two reasons. First, although remote-referee reports are available for all years, their evaluation format is not consistent over time: in 2019-2020, referees assigned separate 1-5 scores for the project and the PI, whereas in 2021-2023 they provided only a categorical overall recommendation ("not recommend," "recommend," "strongly recommend"). This makes the remote-referee assessments difficult to compare quantitatively, both across years and relative to the panel-member scores used in our main analysis. Second, most remote referees review only one proposal, so specifications with remote-referee fixed effects are identified from a much smaller and selected subsample of repeat referees (as reflected in the substantial drop in N). For these reasons, we view the remote-referee results as supplementary and interpret them cautiously; nevertheless, they suggest that external reports can also exhibit homphily, especially for country/citizenship.

Strong Recommendation (Binary, Linear Probability Model)

	**(1)**	**(2)**
Same gender	0.018	-0.001
	(0.019)	(0.978)
	[0.003, 0.032]	[-0.042, 0.041]
Project FE	Yes	Yes
Reviewer gender FE	Yes	
Reviewer FE		Yes
N	31,819	9,533
R^2	0.316	0.791
Within R^2	0.000	0.000
Mean of Y	0.408	0.392
SD of Y	0.491	0.488

Strong Recommendation (Binary, Linear Probability Model)

	(1)	(2)
Same country / same citizenship	0.047	0.169
	(0.019)	(0.000)
	[0.008, 0.087]	[0.080, 0.258]
Same country / different citizenship	0.047	0.079
	(0.009)	(0.042)
	[0.012, 0.082]	[0.003, 0.154]
Different country / same citizenship	0.071	0.131
	(0.000)	(0.000)
	[0.039, 0.103]	[0.065, 0.197]
Project FE	Yes	Yes
Reviewer country/citizenship FE	Yes	
Reviewer FE		Yes
N	30,698	9,084
R^2	0.352	0.796
Within R^2	0.001	0.008
Mean of Y	0.408	0.389
SD of Y	0.491	0.488

5. **The authors note that not all A-rated proposals can be funded and that proposals are therefore ranked, but they do not explain how this ranking is performed in practice. Ranking is often problematic, as panel members may introduce additional, informal criteria, not specified in the regulations, to justify higher or lower ranks.**

We addressed this comment by expanding the description of Step 2. In particular, we now clarify that the ERC does not mandate a specific aggregation rule for turning individual assessments and other inputs (external reports and the interview) into the final within-panel ranking. As a result, panels may differ in how they reconcile disagreement and arrive at a final ordering among A-rated proposals, and the ranking is not necessarily a mechanical function of the individual numeric scores.

6. **Surprisingly, the paper does not include a dedicated Discussion section, but only relatively brief conclusions. The authors should add a Discussion that:**
    - **explicitly reflects on the strengths and weaknesses of the study,**
    - **situates the findings in relation to the growing literature on bias and influence in grant (and possibly journal) peer review (1, 3–8),**

- **discusses the meaning and implications of the results, and**
- **outlines concrete directions for future research.**

We now make sure that the closing section goes beyond merely summarizing results.

Strengths and weaknesses. We now clearly separate what our design identifies causally (individual reviewer scores) from what remains correlational (panel outcomes). In particular, we emphasize as a strength that the individual-score analysis leverages within-proposal and within-reviewer variation to account for unobserved proposal quality and reviewer stringency, while acknowledging that panel-level outcomes cannot use proposal fixed effects because each proposal receives only one panel decision per stage. We also spell out a concrete source of potential confounding factors at the panel level.

Positioning in the literature. We now explicitly relate our findings to recent work on bias and discretion in grant review. We discuss evidence that reviewer characteristics shape assessments (e.g., expertise/strictness and gender-related patterns) and connect our contribution to arguments that discretion is substantial when many proposals are close in quality.

Meaning and implications. We expand the interpretation from "homophily exists" to "outcomes partly depend on who reviews the application," and we explain in more detail how uneven exposure to matching reviewers can create systematic advantages for applicants from larger or more represented research systems, potentially undermining efficiency and the goal of funding the best science.

Future research directions. We now propose specific next steps: evaluating policy interventions empirically, quantifying efficiency/innovation costs, and improving panel-level causal inference via collaborations with agencies and experimental designs.

7. **Relatedly, the paper does not sufficiently discuss policy interventions that could reduce or eliminate homophily bias. The only proposal advanced - excluding reviewers who share the applicant's citizenship or country of work - seems impractical and unrealistic. Other options deserve consideration, including:**
   - **Blinding panel members or reviewers to applicant demographics, at least at the first evaluation stage;**
   - **Partial lotteries for proposals that are clearly fundable but not outstanding, which are especially vulnerable to biased marginal decisions;**
   - **More explicit and tightly defined evaluation criteria, as well as greater diversity of panels in terms of gender, career stage, and background.**

We now address several potential policy interventions that could be implemented. While our data do not allow us to test these interventions directly, we broaden the Discussion to acknowledge that agencies can reduce discretion through process design and that future work should evaluate such interventions.

Reviewer assignment (including panel diversity). We retain the point that assignment rules can reduce exposure to same-citizenship / same-country-of-work matches, but we now present this as one option among several and explicitly note it might not be feasible in all cases. In addition, we now explicitly discuss increasing panel diversity as a complementary lever that can reduce the frequency of such matches and limit the influence of any single in-group perspective in deliberations.

Additional scrutiny near the cutoff / marginal decisions. In direct response to the point that marginal decisions are especially vulnerable to bias, we add a concrete intervention: requiring an additional assessment for proposals near the cutoff, where small score differences are most consequential.

Partial lotteries. We explicitly mention partial lotteries as a mechanism to reduce the influence of potentially biased, low-signal distinctions among closely ranked proposals and cite relevant work.

Blinding / separating identity from project assessment. We add a practical version of blinding that fits grant review realities: separating evaluations into components (e.g., scoring the project using materials that omit PI identifiers where feasible, and scoring the PI separately), thereby limiting identity spillovers rather than assuming perfect anonymization.

More explicit and tightly defined criteria. We now also note that agencies could reduce discretion by making evaluation criteria more explicit and tightly defined, which could limit opportunities for non-merit considerations.

8. **Finally, I disagree with the authors' claim that "while the ERC is international by design, similar dynamics are likely to arise in national funding agencies." In national funding systems, panel members and reviewers are often competitors of the applicants for scarce funding. In such contexts, homophily may instead lead to harsher, not more favourable, assessments.**

We revised the Discussion to explicitly incorporate this caveat. We now note that, even in national systems, citizenship-based matches can remain relevant given international mobility (applicants and reviewers may work in the same country but hold different citizenships). At the same time, we explicitly acknowledge that reviewer–applicant competition may involve different considerations in national settings, so the magnitude, and potentially the direction, of same-citizenship effects could differ from what we observe at the ERC. We leave this as an empirical question for future research.

Response Letter Reviewer 2

We thank the reviewer for the detailed and constructive report and for highlighting both the strengths and the key limitations of our initial submission. We address each suggestion in turn below (we added numbers for easier reference).

1. **Abstract: The reference to "normative" concerns is vague, and if there is sufficient room for elaboration than this adjective should be elaborated so that it is clear what aspect of norms are being referred to, ie according to its clear definition on page 8.**

In the revised manuscript we no longer use the term "normative" in the abstract. More generally, we substantially toned down the earlier "mechanisms" framing and removed statements that could be read as attributing the effects to particular motives. The abstract and main text now focus on the core findings and on what confounder our additional analyses account for.

2. **Figure 3: There is a typo in the panel labels, which are in the order a,c,b,d from top to bottom.**

Thank you for catching this. We corrected the ordering of the panel labels in Figure 3.

3. **Figure 4: Recommend modifying the label "Share different" to "Share of different" to be consistent with text, and also to improve the clarity of the meaning, as without the "of" the label could be interpreted very differently if a reader only were to read the figures.**

We changed the label to "Share of different … panel members" for consistency with the text and to avoid ambiguity in interpretation.

4. **Panel decisions: It should be made more clear in the text that y_i is an (0,1) variable; in which case, it's not clear why a logit or similar binary outcome model was used?**

We clarified the text and notation to make explicit that the panel outcomes are binary. In particular, we now define y more carefully in the description of Equation (2).

We estimate the panel-outcome regressions as a linear probability model. In the text, we now explain that we do so because it (i) facilitates the inclusion and interpretation of multiple fixed effects, (ii) keeps the specification directly comparable to our main analysis of individual numeric scores, and (iii) allows coefficients to be interpreted as changes in probability (percentage points). To address the concern that a non-linear binary model might be more appropriate, we verified that the substantive conclusions are very similar when estimating logit models and calculating average partial effects. For transparency, we report the logit average partial effects below (not included in the paper). They are extremely consistent with the LPM results in sign, magnitude, and inference.

Average partial effects from a Logit Model (p-values in parentheses)

	Assigned A (Step 1)		Assigned A (Step 2)		Funding Offered (Step 2)	
	(1)	(2)	(3)	(4)	(5)	(6)
**Share same country / same citizenship panelists**	**0.264**		**0.164**		**0.135**	
	**(0.000)**		**(0.199)**		**(0.347)**	
**Share same country / different citizenship panelists**	**0.264**		**0.115**		**-0.099**	
	**(0.000)**		**(0.349)**		**(0.462)**	
**Share different country / same citizenship panelists**	**0.155**		**0.154**		**0.193**	
	**(0.009)**		**(0.122)**		**(0.069)**	
**Same country / same citizenship chair**		**0.029**		**0.021**		**0.016**
		**(0.048)**		**(0.369)**		**(0.511)**
**Same country / different citizenship chair**		**0.019**		**0.013**		**0.039**
		**(0.145)**		**(0.565)**		**(0.092)**
**Different country / same citizenship chair**		**0.027**		**0.016**		**0.016**
		**(0.017)**		**(0.492)**		**(0.549)**
**Panel–Scheme–Year FE**	**Yes**	**Yes**	**Yes**	**Yes**	**Yes**	**Yes**
**Applicant country/citizenship FE**	**Yes**	**Yes**	**Yes**	**Yes**	**Yes**	**Yes**
**N**	**36,670**	**36,392**	**11,645**	**11,473**	**11,645**	**11,473**

5. **Discussion: it would be instructive to more specifically discuss recommendations/reflections regarding the multi-stage format of the ERC system. What are the pros/cons of this format associated with the findings; and what steps could be made to improve the fidelity of the peer review system – e.g. excluding same/country-citizenship matches among reviewer in Step 1, at the cost of reducing the sample size for determining the Step 1 proposal grade.**

We agree that the interpretation of our findings depends in part on the ERC's two-step design, and we revised the Discussion to speak to this more directly.

First, we now add an explicit paragraph reflecting on the pros and cons. We note that a potential advantage of a multi-stage process is that later-step information can, in principle, correct idiosyncratic judgments from earlier reviews. At the same time, we emphasize that Step 1 is a gatekeeping stage: even modest identity-related differences in individual scores can affect which proposals advance and thus who benefits from the more information-rich second stage.

Second, we expanded the Discussion's policy paragraph to connect interventions to the evaluation procedure. In particular, we now discuss reviewer assignment policies that could reduce exposure to same-citizenship and same-country-of-work matches (while noting that such restrictions may not be feasible in all cases), which directly speaks to your example of targeting Step 1 assignments. We also discuss additional measures (e.g., additional assessment for proposals near the cutoff, partial lotteries for borderline cases, and separating project from PI assessments where feasible).

Response Letter Reviewer 3

We thank the reviewer for the detailed and constructive report and for highlighting both the strengths and the key limitations of our initial submission. We address each suggestion in turn below (we added numbers for easier reference).

1. **I do not find the mechanisms explaining why evaluators rate academics working in their own country or with the same citizenship more highly convincing. First, a combination of factors such as information, reciprocity, or in-group loyalty might be at play. Such an interplay cannot be ruled out. Moreover, at the panel level, homophily appears only in step one of the evaluation process, but not in the second. What drives this difference between the panel and evaluators? And why does a homophily bias emerge in the panel at step 1, but not in step 2? How does this align with the proposed mechanisms? The suggested mechanisms also raise the question of why these considerations do not seem to apply to women. Overall, I find the discussion of mechanisms raises more questions than it answers, and as it is only addressed for evaluators, it does not seem very meaningful. However, I also don't think much can be done to clarify the mechanisms with the data available. Overall, I would not mind if the authors removed the discussion of mechanisms altogether. If this is not the preferred option, I would suggest (i) providing a discussion of mechanisms related to homophily for gender and country/citizenship, (ii) explaining why differences exist between the two categories– gender and country/citizenship, and (iii) highlighting why we see disparities in homophily between evaluators, panel 1, and panel 2.**

   In the revised manuscript, we removed the mechanisms discussion and no longer speculate about why the effects arise. Instead, we reframe this part of the results as assessing whether the estimated same-country and same-citizenship effects in individual reviews could be confounded by (i) observable proxies for shared tastes/topic proximity or (ii) informational advantages.

   First, we control for semantic similarity and coauthorship proximity, and, in response to a suggestion of Referee 1, we additionally add a control for ERC keyword topic match. Second, we retain the score-distribution analysis; the effects are concentrated at the top of the score distribution, with little corresponding increase in low-end scores, making it less likely that an unobservable confounder reflecting information advantages affects the estimates.

2. **I find the discussion of same-country and same-citizenship effects in panel decisions underdeveloped. First, for step 1 and step 2, no tables are referenced, making it challenging to evaluate what is going on. Which tables show these effects?**

In the revised manuscript, we now explicitly reference the table that reports the full set of panel-level estimates for both Step 1 and Step 2. Specifically, we added a pointer to Supplementary Table 27 when introducing Panels C and D of Fig. 4 and again when discussing Step-2 outcomes.

In addition, we expanded the summary of the panel-decision results to make the Step-1 versus Step-2 patterns easier to follow: we clarify the three match categories (only citizenship, only country of work, or both), explicitly state that Panels C and D report estimates for Assigned A (Step 1), Assigned A (Step 2), and Funding Offered (Step 2), and briefly highlight the contrast between statistically significant Step-1 associations and smaller, statistically insignificant Step-2 associations.

3. **The part I found most unclear was the discussion of homophily-affected selection in step 2. The argument is that, given homophily in previous stages, homophily may ultimately still affect the funding decision even though, at the step 2 panel, there is no homophily present. The authors therefore calculate the probability of receiving funding conditional on reaching Step 2, multiplied by the probability of reaching Step 2. They then take the**

derivative and obtain a term in the methodology section, but it is not clear where this term comes from. This needs to be explained much better in the methodology section. And where do the numbers in the main section come from regarding the probabilities come from? Can they be matched to a table?

We rewrote the relevant Methods subsection to explain more transparently how the unconditional funding probability can be expressed as the product of the probability of advancing to Step 2 and the probability of being funded conditional on advancing, and that the overall association of panel–applicant matches with funding therefore combines Step-1 and Step-2 associations.

Second, we added an explicit numerical example in the main text illustrating how the combined effect is constructed from the Step-1 and Step-2 components.

Third, we added a new Table 1 and now reference it in the main text. The table reports the Step-1 and Step-2 coefficients and the fitted probabilities used for the calculation, alongside the resulting combined effects and their inference, so that readers can directly match the main-text quantities to a single table.

4. **Economic significance: What is the overall economic significance of homophily for the probability of receiving funding? There should be a discussion of how large these effects are. At this stage, it is not obvious that there is a bias in the ultimate funding decision or how economically meaningful it is. It would also be useful to understand how the probability of funding differs for applicants from a large country versus a small country (for example, Germany versus Luxembourg), to gain some intuition.**

In the revised manuscript, we expanded the Discussion to make the economic significance of the estimated homophily patterns more transparent. We translate differences in panel composition into implied differences in unconditional funding probabilities using the average partial effects reported in Table 1, and we emphasize that even modest homophily in individual scores can matter because Step 1 is a gatekeeping stage.

To provide intuition for large versus small/underrepresented countries, we contrast applicants from the five most represented applicant countries (Germany, the UK, France, Italy, and Spain) with applicants from all other countries. We also added Table 2, which reports average match shares by group and combines them with the estimated average partial effects to obtain predicted homophily-related differences in unconditional funding probabilities. This implies an expected homophily-related increase of 1.7 p.p. for applicants from the top-five countries versus 0.8 p.p. for applicants from all other countries, i.e., a gap of 0.9 p.p.

Response Letter Reviewer 1

We thank the reviewer for the additional comments regarding our revision. We address each comment below.

1. **The authors now present new results in the Discussion section and I wondered whether they should be moved to the Results.**

In the revised manuscript we have moved the calculation on the benefits of homophily for applicants from more vs. less represented countries to the end of the subsection “Same-country and same-citizenship homophily in panel decisions”.

2. **They use the term ‘no evidence’ but a p value >0.05 does not mean there is no evidence. Suggest you rephrase, for example ‘little evidence’ or ‘no strong evidence’.**

We have revised the wording accordingly. In particular, for the panel-level gender results, where one estimate is significant at the 10% level and points in the opposite direction, we now write: “While there is no robust evidence of homophily by panels in terms of gender - and even marginally significant evidence to the contrary - .”

Response Letter Reviewer 2

We thank the reviewer for the additional comment regarding our revision, which we address below.

1. **While I follow your argument that using a linear probability model facilitates consistent comparison with the other models based upon numerical scores, it will still not sit well with those familiar with the potential pitfalls of applying linear models to binary dependent variables. Hence, my only remaining recommendation is to include the Logit model results in the supplementary information.**

In the revised manuscript, we report the average marginal effects from the Logit models in the Supplementary Information (Supplementary Table 33, repeated below). We also identified a very minor error in the calculation of the p-values for the average marginal effects reported in the table included in our previous response letter. This error does not affect the estimated average marginal effects or their substantive interpretation, and only leads to negligible changes in the reported p values in a small number of cases. Note that we moved this paragraph to the end of the results section, so that this robustness check follows the preferred specification.

Average partial effects from Logit Model (p-values in parentheses)

	Assigned A (Step 1)		Assigned A (Step 2)		Funding Offered (Step 2)	
	(1)	(2)	(3)	(4)	(5)	(6)
Share of same country/ same citz. panelists	0.264		0.164		0.135	
	(0.000)		(0.195)		(0.348)	
	[0.124, 0.404]		[−0.084, 0.412]		[−0.147, 0.417]	
Share of same country/ diff. citz. panelists	0.264		0.115		−0.099	
	(0.001)		(0.346)		(0.461)	
	[0.114, 0.415]		[−0.125, 0.355]		[−0.363, 0.164]	
Share of diff. country/ same citz. panelists	0.155		0.154		0.193	
	(0.009)		(0.119)		(0.069)	
	[0.038, 0.271]		[−0.040, 0.347]		[−0.015, 0.402]	
Same country/ same citz. chair		0.029		0.021		0.016
		(0.048)		(0.368)		(0.511)
		[0.000, 0.058]		[−0.025, 0.068]		[−0.032, 0.064]
Same country/ diff. citz. chair		0.019		0.013		0.039
		(0.145)		(0.565)		(0.092)
		[−0.007, 0.045]		[−0.031, 0.057]		[−0.006, 0.084]
Diff. country/ same citz. chair		0.027		0.016		0.016
		(0.017)		(0.492)		(0.549)
		[0.005, 0.050]		[−0.029, 0.061]		[−0.035, 0.066]

Response Letter Reviewer 3

We thank the reviewer for the additional comments regarding our revision. We address each comment below.

1. **You emphasize that your contribution is to provide a causal estimate of homophily. This is correct for the evaluation in the first stage of the ERC process, but not for the overall success rate. It would be good to be precise on this.**

We agree that the causal interpretation applies to individual reviewer scores, whereas the analysis of panel decisions and funding outcomes is correlational. To make this distinction clearer, we revised the contribution paragraph in the introduction to state explicitly that our causal estimates concern only the individual reviews.

2. **P.11 lines 266-268: it would be good to state in one short sentence how the results of the robustness check compare.**

We have added a brief summary of the robustness check results to the main text. Specifically, we now note that using only assigned reviewers yields the same main conclusion: the strongest and most robust positive association is found when applicants and evaluators share both country of work and citizenship, while the country-only and citizenship-only associations are less stable. Note that we moved this paragraph to the end of the results section, so that this robustness check follows the preferred specification.
